# New species and records of *Trichoderma* isolated as mycoparasites and endophytes from cultivated and wild coffee in Africa

**DOI:** 10.1038/s41598-021-84111-1

**Published:** 2021-03-11

**Authors:** María del Carmen H. Rodríguez, Harry C. Evans, Lucas M. de Abreu, Davi M. de Macedo, Miraine K. Ndacnou, Kifle B. Bekele, Robert W. Barreto

**Affiliations:** 1 Departamento de Fitopatologia, Universidade Federal de Viçosa, Viçosa, MG 36570-900 Brazil; 2 CAB International, Bakeham Lane, Egham, Surrey TW20 9TY UK; 3 IRAD-Institut de Recheche Agricole pour le Developpement, BP 2067 Yaoundé, Cameroon; 4 Department of Horticulture and Plant Science, College of Agriculture and Veterinary Medicine, Jimma University, P.O. Box 397, Jimma, Ethiopia; 5 Ethiopian Institute of Agriculture Research, P.O. Box 192, Jimma, Ethiopia

**Keywords:** Microbiology, Plant sciences

## Abstract

A survey for species of the genus *Trichoderma* occurring as endophytes of *Coffea,* and as mycoparasites of coffee rusts (*Hemileia*), was undertaken in Africa; concentrating on Cameroon and Ethiopia. Ninety-four isolates of *Trichoderma* were obtained during this study: 76 as endophytes of healthy leaves, stems and berries and, 18 directly from colonized rust pustules. A phylogenetic analysis of all isolates used a combination of three genes: translation elongation factor-1α (*tef1*), *rpb2* and *cal* for selected isolates. GCPSR criteria were used for the recognition of species; supported by morphological and cultural characters. The results reveal a previously unrecorded diversity of *Trichoderma* species endophytic in both wild and cultivated *Coffea*, and mycoparasitic on *Hemileia* rusts. Sixteen species were delimited, including four novel taxa which are described herein: *T. botryosum*, *T. caeruloviride*, *T. lentissimum* and *T. pseudopyramidale*. Two of these new species, *T*. *botryosum* and *T*. *pseudopyramidale*, constituted over 60% of the total isolations, predominantly from wild *C*. *arabica* in Ethiopian cloud forest. In sharp contrast, not a single isolate of *Trichoderma* was obtained using the same isolation protocol during a survey of coffee in four Brazilian states, suggesting the existence of a ‘*Trichoderma* void’ in the endophyte mycobiota of coffee outside of Africa. The potential use of these African *Trichoderma* isolates in classical biological control, either as endophytic bodyguards—to protect coffee plants from *Hemileia vastatrix*, the fungus causing coffee leaf rust (CLR)—or to reduce its impact through mycoparasitism, is discussed, with reference to the on-going CLR crisis in Central America.

## Introduction

Species of the ascomycete genus *Trichoderma* (*Hypocreales*: *Hypocreaceae*) are widely distributed in different environments and have a variety of biological activities^1^. In the last two decades various studies have investigated the diversity and taxonomy of *Trichoderma* and numerous novel species have emerged using DNA sequence data^2–5^. However, despite the various surveys aimed at covering the diversity of this genus, such studies have been concentrated mostly in Asia, Europe and the Americas^6–13^. In contrast, until now, Africa has been poorly covered in terms of assessing the diversity of *Trichoderma*, with the exception of some studies involving specific regions or ecological niches, such as soil in South Africa^14^. In the case of *Trichoderma* occurring as endophytes in *Coffea*, there is a single study covering species isolated from the rhizosphere of *C*. *arabica* in Ethiopia^15^.

Fungi belonging to the genus *Trichoderma* have a recognized role as decomposers^16,17^ and for a long time they were considered to be soil saprotrophs of little practical relevance^18,19^. Currently, it is widely accepted that such a generalization was erroneous and many species of *Trichoderma* are now recognized as mycoparasites, as well as endophytes of woody plants^18,20–24^. The endophytic interaction between *Trichoderma* and their host-plants is intimate and may be complex, involving many steps at each level from direct contact to internal colonization of tissues^19^. Endophytic *Trichoderma* may behave as innocuous commensals or as true symbionts; stimulating the plant’s defence system in various ways: inducing host resistance to plant pests; promoting tolerance to abiotic stresses; increasing plant growth and photosynthetic capability; and, contributing towards the solubilization of nutrients for the host plant’s benefit^25–33^.

Studies on *Trichoderma* as endophytes in perennial crop plants, particularly in their original wild to semi-wild situations, have revealed a considerable diversity of species—including a number of novel taxa—especially when compared with the same crops in cultivation. Notable examples are cacao, *Theobroma cacao*^20,34–38^ and rubber, *Hevea brasiliensis*^8,39–41^ in their native Amazonian ranges.

Members of *Trichoderma* compete naturally in the wild with other groups of fungi to occupy niches and obtain nutrients and are capable of producing a range of secondary metabolites, including antibiotics and mycotoxins^18,42,43^. Another characteristic of *Trichoderma* is the mycoparasitic ability of certain species which has led them to be considered as potential tools for the control of plant pathogenic fungi^21^. There are several practical examples of the commercial application of mycoparasitic *Trichoderma*: notably, that of *T. stromaticum* for control of *Moniliophthora perniciosa*—the causal agent of witches’ broom disease of cacao—the most important disease of the crop in the Neotropics. This mycoparasite colonizes the necrotic brooms of diseased plants, as well as the agaric fruit bodies of the fungus, decreasing inoculum production^44^, and it has also been reported to be a common endophyte in healthy cacao trees^45^. A product based on *T. stromaticum* (Tricovab) has been distributed to farmers in southern Bahia (Brazil) for a number of years^5,46^, and now forms part of an integrated management strategy^47^*.*

*Trichoderma* species, such as *T*. *harzianum*, can colonize and degrade resistant structures (sclerotia) of other plant pathogenic fungi^48,49^, and have been mass-produced and used as commercial bio-fungicides^5,50^. Although the known diversity of *Trichoderma* is already high, with more than 200 species names recognized, based on molecular phylogeny^51,52^, most research on mycoparasitism has been undertaken with only a few of these species, including: *T. harzianum *sensu lato, *T. atroviride*, *T. virens*, *T. asperellum* and *T. asperelloides*^49^, whilst mycoparasitism of rust fungi by *Trichoderma* has been little studied. The potential of *Trichoderma* as a tool for the management of plant diseases is now widely recognized, although this approach has virtually been untapped for many diseases of tropical perennial crops. The main aim of this study was to collect, isolate and identify members of the genus *Trichoderma* from *Coffea* species and their associated *Hemileia* rusts in their centres of origin in Africa, with the long-term objective of assessing their potential as biocontrol agents of coffee leaf rust (CLR) caused by *Hemileia vastatrix* an increasing constraint to coffee production in the Americas^53^.

## Results

### Phylogenetic analyses and GCPRS

**Figure 1:**
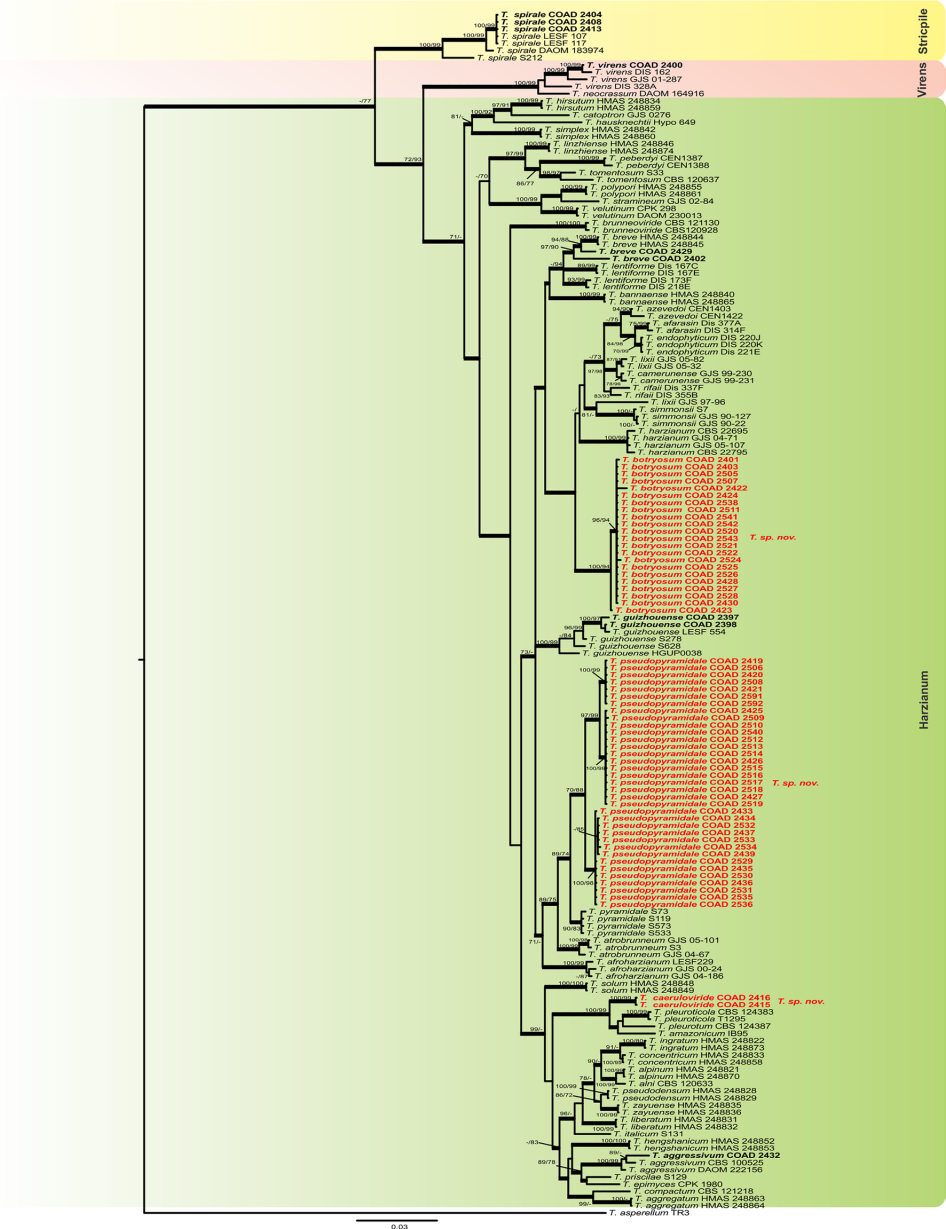
Bayesian phylogenetic tree of clades *Harzianum*, *Strictipile* and *Virens*. The tree was based on a concatenated *tef1* and *rpb2* sequence dataset. Bootstrap values (≥ 70%) of the ML and MP analyses, as well as posterior probability scores (≥ 0.9) from a Bayesian analysis of the same dataset, are indicated at well supported nodes together with thickened branches. The isolates belonging to known species, obtained in this study, are in bold. Isolates of new species, described in this study, are in bold red. The tree was rooted with *Trichoderma asperellum* (TR3). The phylogenetic tree was edited using Inkscape 1.0 (https://inkscape.org/pt-br/).

**Figure 2:**
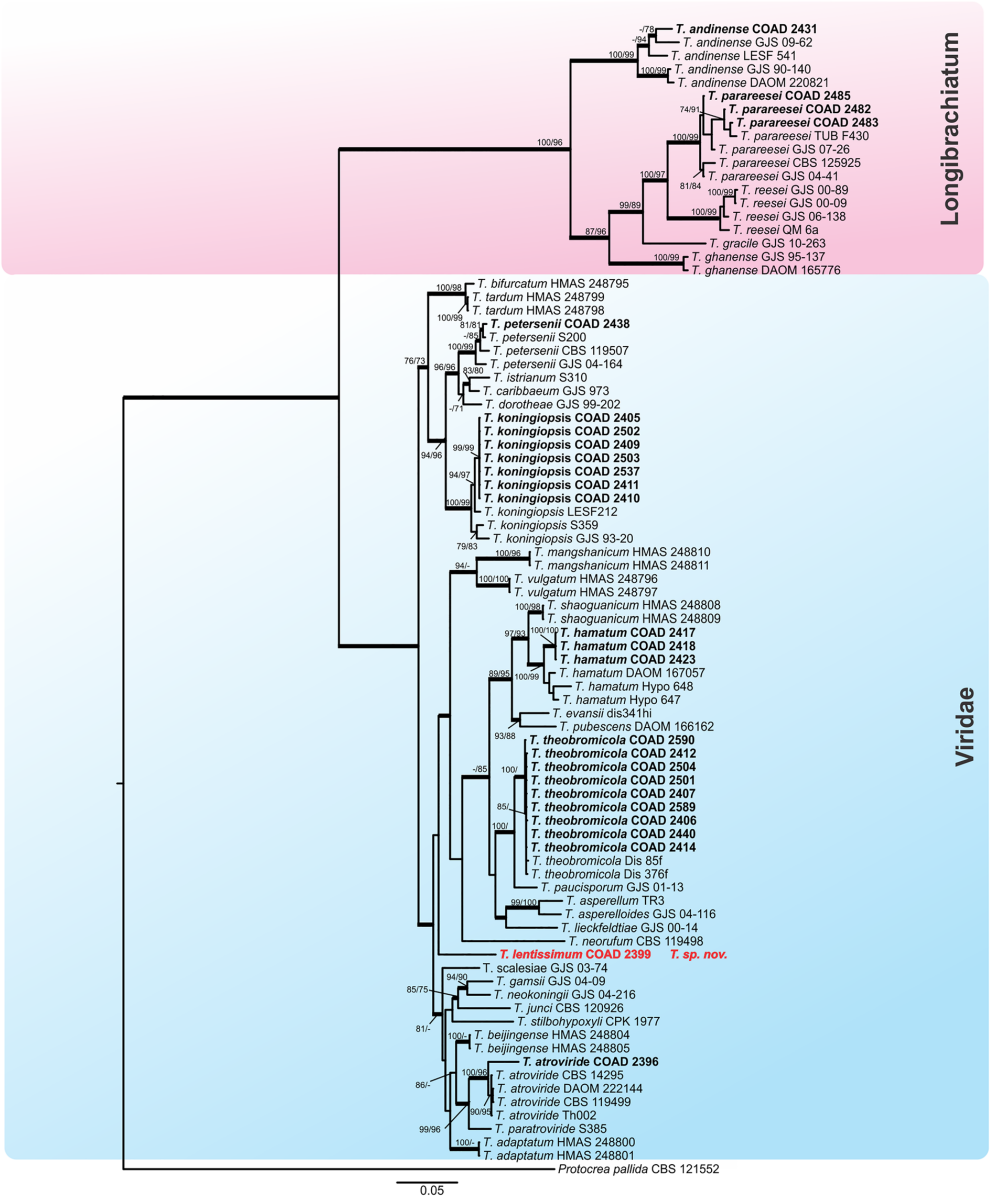
Bayesian phylogenetic tree of clades *Longibrachiatum* and *Viride*. The tree was based on a concatenated tef1 and rpb2 sequence dataset. Bootstrap values (≥ 70%) of the ML and MP analyses, as well as posterior probability scores (≥ 0.9) from a Bayesian analysis of the same dataset, are indicated at well supported nodes together with thickened branches. The isolates belonging to known species, obtained in this study, are in bold. Isolates of new species, described in this study, are in bold red. The tree was rooted with *Protocrea pallida* (CBS 121552). The phylogenetic tree was edited using Inkscape 1.0 (https://inkscape.org/pt-br/).

A total of 94 *Trichoderma* isolates were obtained during this survey, 76 as endophytes in *Coffea* spp. and 18 as mycoparasites on coffee rusts (Table 1). The combined data set indicated that the 94 *Trichoderma* strains grouped into 16 highly supported monophyletic groups (Figs. 1 and 2). The concatenated trees generated in BI, ML and MP analyses shared a similar topology, providing high support to the final trees. Phylogenetic trees and DNA sequence alignment data are available from TreeBase (study S27041).

**Table 1:** *Trichoderma* strains obtained in the survey and used in the phylogenetic analyses, with their corresponding geographic origin, host and tissue source.

Taxon	Isolate	Country	Substrate	Genbank accession numbers		
				*tef*	*rpb2*	*cal*
*T. aggressivum*	COAD 2432	Kenya	*Hemileia* sp. Mycoparasite	MK044156	MK044249	–
*T. andinense*	COAD 2431	Brazil	*Hemileia vastatrix*, Mycoparasite	MK044155	MK044248	–
*T. atroviride*	COAD 2396	Kenya	Leaf, *Coffea* sp. Endophyte	MK044083	MK044177	–
*T. botryosum* sp. nov.	**COAD 2422**	**Ethiopia**	**Berry, Coffea arabica Endophyte**	**MK044119**	**MK044212**	–
*T. botryosum * sp. nov.	COAD 2401	Cameroon	Stem, *Coffea canephora* Endophyte	MK044088	MK044181	–
*T. botryosum * sp. nov.	COAD 2403	Cameroon	Stem, *Coffea arabica* Endophyte	MK044090	MK044183	–
*T. botryosum * sp. nov.	COAD 2505	Ethiopia	Stem, *Coffea arabica* Endophyte	MK044112	MK044205	
*T. botryosum * sp. nov.	COAD 2507	Ethiopia	Berry, *Coffea arabica* Endophyte	MK044116	MK044209	–
*T. botryosum * sp. nov.	COAD 2424	Ethiopia	Leaf, *Coffea arabica* Endophyte	MK044121	MK044214	–
*T. botryosum * sp. nov.	COAD 2538	Ethiopia	Leaf, *Coffea arabica* Endophyte	MK044122	MK044215	–
*T. botryosum * sp. nov.	COAD 2511	Ethiopia	Leaf, *Coffea arabica* Endophyte	MK044126	MK044219	–
*T. botryosum * sp. nov.	COAD 2541	Ethiopia	Stem, *Coffea arabica* Endophyte	MK044138	MK044231	–
*T. botryosum * sp. nov.	COAD 2542	Ethiopia	Stem, *Coffea arabica* Endophyte	MK044139	MK044232	–
*T. botryosum * sp. nov.	COAD 2520	Ethiopia	Stem, *Coffea arabica* Endophyte	MK044140	MK044233	–
*T. botryosum * sp. nov.	COAD 2543	Ethiopia	Stem, *Coffea arabica* Endophyte	MK044141	MK044234	–
*T. botryosum * sp. nov.	COAD 2521	Ethiopia	Stem, *Coffea arabica* Endophyte	MK044142	MK044235	–
*T. botryosum * sp. nov.	COAD 2522	Ethiopia	Stem, *Coffea arabica* Endophyte	MK044143	MK044236	–
*T. botryosum * sp. nov.	COAD 2423	Ethiopia	Stem, *Coffea arabica* Endophyte	MK044144	MK044237	–
*T. botryosum * sp. nov.	COAD 2524	Ethiopia	stem, *Coffea arabica* Endophyte	MK044145	MK044238	–
*T. botryosum * sp. nov.	COAD 2525	Ethiopia	Stem, *Coffea arabica* Endophyte	MK044146	MK044239	–
*T. botryosum * sp. nov.	COAD 2526	Ethiopia	Stem, *Coffea arabica* Endophyte	MK044147	MK044240	–
*T. botryosum * sp. nov.	COAD 2428	Ethiopia	Berry, *Coffea arabica* Endophyte	MK044148	MK044241	–
*T. botryosum * sp. nov.	COAD 2527	Ethiopia	Leaf, *Coffea arabica* Endophyte	MK044149	MK044242	–
*T. botryosum * sp. nov.	COAD 2528	Ethiopia	Leaf, *Coffea arabica* Endophyte	MK044151	MK044244	–
*T. botryosum * sp. nov.	COAD 2430	Ethiopia	Leaf, *Coffea arabica* Endophyte	MK044152	MK044245	–
*T. breve*	COAD 2402	Cameroon	Stem, *Coffea canephora* Endophyte	MK044089	MK044182	–
*T. breve*	COAD 2429	Ethiopia	Berry, *Coffea arabica* Endophyte	MK044150	MK044243	–
*T. caeruloviride * sp. nov.	COAD 2416	Ethiopia	Berry, *Coffea arabica* Endophyte	MK044108	MK044201	–
*T. caeruloviride * sp. nov.	**COAD 2415**	**Ethiopia**	**Berry, Coffea arabica Endophyte**	**MK044109**	**MK044202**	–
*T. guizhouense*	COAD 2397	Kenya	stem, *Coffea* sp Endophyte	MK044084	MK044176	–
*T. guizhouense*	COAD 2398	Kenya	Stem, *Coffea* sp. Endophyte	MK044085	MK044178	–
*T. hamatum*	COAD 2417	Ethiopia	Stem, *Coffea arabica* Endophyte	MK044110	MK044203	–
*T. hamatum*	COAD 2418	Ethiopia	Stem, *Coffea arabica* Endophyte	MK044111	MK044204	–
*T. hamatum*	COAD 2423	Ethiopia	Berry, *Coffea arabica* Endophyte	MK044120	MK044213	–
*T. koningiopsis*	COAD 2405	Cameroon	Leaf, *Coffea canephora* Endophyte	MK044092	MK044185	–
*T. koningiopsis*	COAD 2502	Cameroon	Leaf, *Coffea canephora* Endophyte	MK044097	MK044190	–
*T. koningiopsis*	COAD 2537	Cameroon	Leaf, *Coffea canephora* Endophyte	MK044098	MK044191	–
*T. koningiopsis*	COAD 2409	Cameroon	Stem, *Coffea canephora* Endophyte	MK044099	MK044192	–
*T. koningiopsis*	COAD 2503	Cameroon	Leaf, *Coffea canephora* Endophyte	MK044100	MK044193	–
*T. koningiopsis*	COAD 2410	Cameroon	Leaf, *Coffea canephora* Endophyte	MK044101	MK044194	–
*T. koningiopsis*	COAD 2411	Cameroon	Leaf, *Coffea canephora* Endophyte	MK044102	MK044195	–
*T. lentissimum * sp. nov.	**COAD 2399**	**Kenya**	**Stem, Coffea cf. arabica Endophyte**^b^	**MK044086**	**MK044179**	–
*T. parareesei*	COAD 2485	Ethiopia	*Hemileia* sp. Mycoparasite^a^	MK044082	MK044265	–
*T. parareesei*	COAD 2482	Ethiopia	Stem, *Coffea arabica* Endophyte	MK044153	MK044246	–
*T. parareesei*	COAD 2483	Ethiopia	Stem, *Coffea arabica* Endophyte	MK044154	MK044247	–
*T. petersenii*	COAD 2438	Ethiopia	*Hemileia* sp. Mycoparasite^a^	MK044168	MK044261	–
*T. pseudopyramidale * sp. nov.	COAD 2419	Ethiopia	Stem, *Coffea arabica* Endophyte	MK044113	MK044206	MK084875
*T. pseudopyramidale * sp. nov.	COAD 2506	Ethiopia	Stem, *Coffea arabica* Endophyte	MK044114	MK044207	–
*T. pseudopyramidale * sp. nov.	COAD 2420	Ethiopia	Stem, *Coffea arabica* Endophyte	MK044115	MK044208	MK084874
*T. pseudopyramidale * sp. nov.	COAD 2508	Ethiopia	Leaf, *Coffea arabica* Endophyte	MK044117	MK044210	–
*T. pseudopyramidale * sp. nov.	COAD 2421	Ethiopia	Leaf, *Coffea arabica* Endophyte	MK044118	MK044211	MK084873
*T. pseudopyramidale * sp. nov.	COAD 2425	Ethiopia	Leaf, *Coffea arabica* Endophyte	MK044123	MK044216	MK084871
*T. pseudopyramidale * sp. nov.	COAD 2509	Ethiopia	Leaf, *Coffea arabica* Endophyte	MK044124	MK044217	–
*T. pseudopyramidale * sp. nov.	COAD 2510	Ethiopia	Leaf, *Coffea arabica* Endophyte	MK044125	MK044218	–
*T. pseudopyramidale * sp. nov.	COAD 2540	Ethiopia	Leaf, *Coffea arabica* Endophyte	MK044127	MK044220	–
*T. pseudopyramidale * sp. nov.	COAD 2512	Ethiopia	Leaf, *Coffea arabica* Endophyte	MK044128	MK044221	–
*T. pseudopyramidale * sp. nov.	COAD 2513	Ethiopia	Leaf, Coffea *arabica* Endophyte	MK044129	MK044222	–
*T. pseudopyramidale * sp. nov.	COAD 2514	Ethiopia	Leaf, *Coffea arabica* Endophyte	MK044130	MK044223	–
*T. pseudopyramidale * sp. nov.	**COAD 2426**	**Ethiopia**	**Leaf, Coffea arabica Endophyte**	**MK044131**	**MK044224**	**MK084870**
*T. pseudopyramidale * sp. nov.	COAD 2515	Ethiopia	Leaf, *Coffea arabica* Endophyte	MK044132	MK044225	–
*T. pseudopyramidale * sp. nov.	COAD 2516	Ethiopia	Leaf, *Coffea arabica* Endophyte	MK044133	MK044226	–
*T. pseudopyramidale * sp. nov.	COAD 2517	Ethiopia	Leaf, *Coffea arabica* Endophyte	MK044134	MK044227	–
*T. pseudopyramidale * sp. nov.	COAD 2518	Ethiopia	Leaf, *Coffea arabica* Endophyte	MK044135	MK044228	–
*T. pseudopyramidale * sp. nov.	COAD 2427	Ethiopia	Leaf, *Coffea arabica* Endophyte	MK044136	MK044229	MK084872
*T. pseudopyramidale * sp. nov.	COAD 2519	Ethiopia	Leaf, *Coffea arabica* Endophyte	MK044137	MK044230	–
*T. pseudopyramidale * sp. nov.	COAD 2433	Cameroon	*Hemileia* sp. Mycoparasite	MK044157	MK044250	MK084869
*T. pseudopyramidale * sp. nov.	COAD 2434	Ethiopia	*Hemileia* sp. Mycoparasite^a^	MK044158	MK044251	MK084868
*T. pseudopyramidale * sp. nov.	COAD 2529	Ethiopia	*Hemileia* sp. Mycoparasite^a^	MK044159	MK044252	–
*T. pseudopyramidale * sp. nov.	COAD 2435	Ethiopia	*Hemileia* sp. Mycoparasite^a^	MK044160	MK044253	MK084867
*T. pseudopyramidale * sp. nov.	COAD 2530	Ethiopia	*Hemileia* sp. Mycoparasite^a^	MK044161	MK044254	–
*T. pseudopyramidale * sp. nov.	COAD 2436	Ethiopia	*Hemileia* sp. Mycoparasite^a^	MK044162	MK044255	MK084865
*T. pseudopyramidale * sp. nov.	COAD 2531	Ethiopia	*Hemileia* sp. Mycoparasite^a^	MK044163	MK044256	–
*T. pseudopyramidale * sp. nov.	COAD 2532	Ethiopia	*Hemileia* sp. Mycoparasite^a^	MK044164	MK044257	–
*T. pseudopyramidale * sp. nov.	COAD 2437	Ethiopia	*Hemileia* sp. Mycoparasite^a^	MK044165	MK044258	MK084866
*T. pseudopyramidale * sp. nov.	COAD 2533	Ethiopia	*Hemileia* sp. Mycoparasite^a^	MK044166	MK044259	–
*T. pseudopyramidale * sp. nov.	COAD 2534	Ethiopia	*Hemileia* sp. Mycoparasite^a^	MK044167	MK044260	–
*T. pseudopyramidale * sp. nov.	COAD 2535	Ethiopia	*Hemileia* sp. Mycoparasite^a^	MK044169	MK044262	–
*T. pseudopyramidale* sp. nov.	COAD 2536	Ethiopia	*Hemileia* sp. Mycoparasite^a^	MK044170	MK044263	–
*T. pseudopyramidale* sp. nov.	COAD 2439	Ethiopia	*Hemileia* sp. Mycoparasite^a^	MK044171	MK044264	MK084864
*T. pseudopyramidale* sp. nov.	COAD 2591	Ethiopia	Stem, *Coffea arabica* L. Endophyte	MK044174	MK044268	–
*T. pseudopyramidale* sp. nov.	COAD 2592	Ethiopia	Stem, *Coffea arabica* L. Endophyte	MK044175	MK044269	–
*T. spirale*	COAD 2404	Cameroon	Stem, *Coffea canephora* Endophyte	MK044091	MK044184	–
*T. spirale*	COAD 2408	Cameroon	Stem, *Coffea canephora* Endophyte	MK044096	MK044189	–
*T. spirale*	COAD 2413	Cameroon	Stem, *Coffea canephora* Endophyte	MK044105	MK044198	–
*T. theobromicola*	COAD 2406	Cameroon	Stem, *Coffea canephora* Endophyte	MK044093	MK044186	–
*T. theobromicola*	COAD 2407	Cameroon	Stem, *Coffea canephora* Endophyte	MK044094	MK044187	–
*T. theobromicola*	COAD 2501	Cameroon	Stem, *Coffea canephora* Endophyte	MK044095	MK044188	–
*T. theobromicola*	COAD 2504	Cameroon	Stem, *Coffea canephora* Endophyte	MK044103	MK044196	–
*T. theobromicola*	COAD 2412	Cameroon	Stem, *Coffea canephora* Endophyte	MK044104	MK044197	–
*T. theobromicola*	COAD 2440	Cameroon	Stem, *Coffea canephora* Endophyte	MK044106	MK044199	–
*T. theobromicola*	COAD 2414	Cameroon	Stem, *Coffea canephora* Endophyte	MK044107	MK044200	–
*T. theobromicola*	COAD 2589	Cameroon	Stem, *Coffea canephora* Endophyte	MK044172	MK044266	–
*T. theobromicola*	COAD 2590	Cameroon	Stem, *Coffea canephora* Endophyte	MK044173	MK044267	–
*T. virens*	COAD 2400	Cameroon	Stem, *Coffea brevipes* Endophyte	MK044087	MK044180	–

*Trichoderma* strains isolated during this study. Ex-type strains are indicated in bold.

^a^Close to *H*. *coffeicola*, but currently being assessed as a new species of *Hemileia* (Authors, unpublished).

^b^Identified as a wild and geographically-isolated population of *Coffea arabica*, common in the understorey forest^54^. Kew Herbarium (Herb K) records reflect uncertainty about its true identity; botanical specimens from present survey deposited in Herb K.

**Figure 3:**
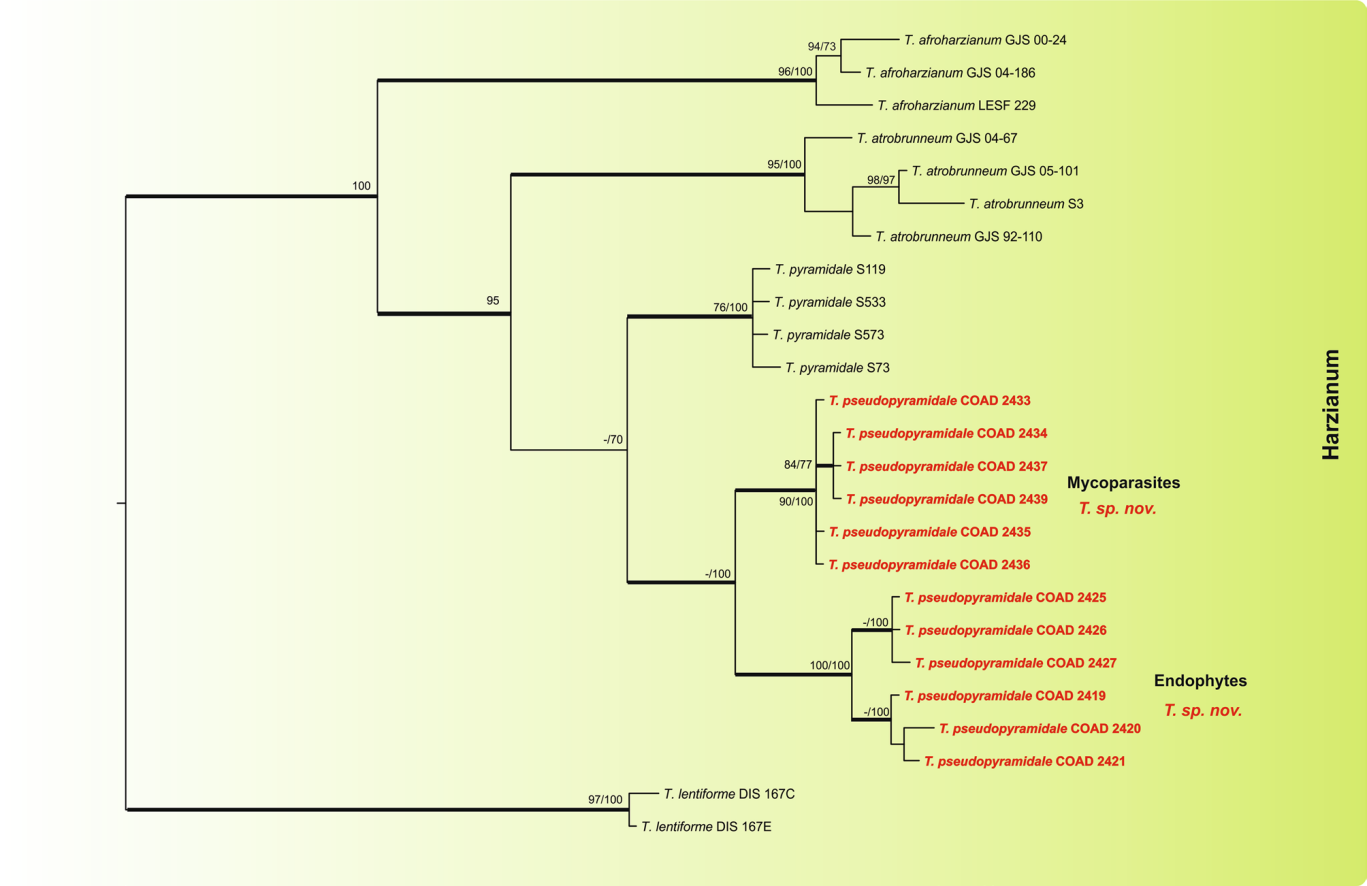
Bayesian phylogenetic tree of *T. pyramidale* and *T. pseudopyramidale* sp. nov. The tree was based on a concatenated tef1, rpb2 and cal sequence dataset. Bootstrap values (≥ 70%) of the ML and MP analyses, as well as posterior probability scores (≥ 0.9) from a Bayesian analysis of the same dataset, are indicated at well supported nodes together with thickened branches. The isolates belonging to known species, obtained in this study, are in bold. Isolates of new species, described in this study, are in bold red. The tree was rooted with *Trichoderma lentiforme* (DIS 167C and DIS 167E). The phylogenetic tree was edited using Inkscape 1.0 (https://inkscape.org/pt-br/).

**Figure 4:**
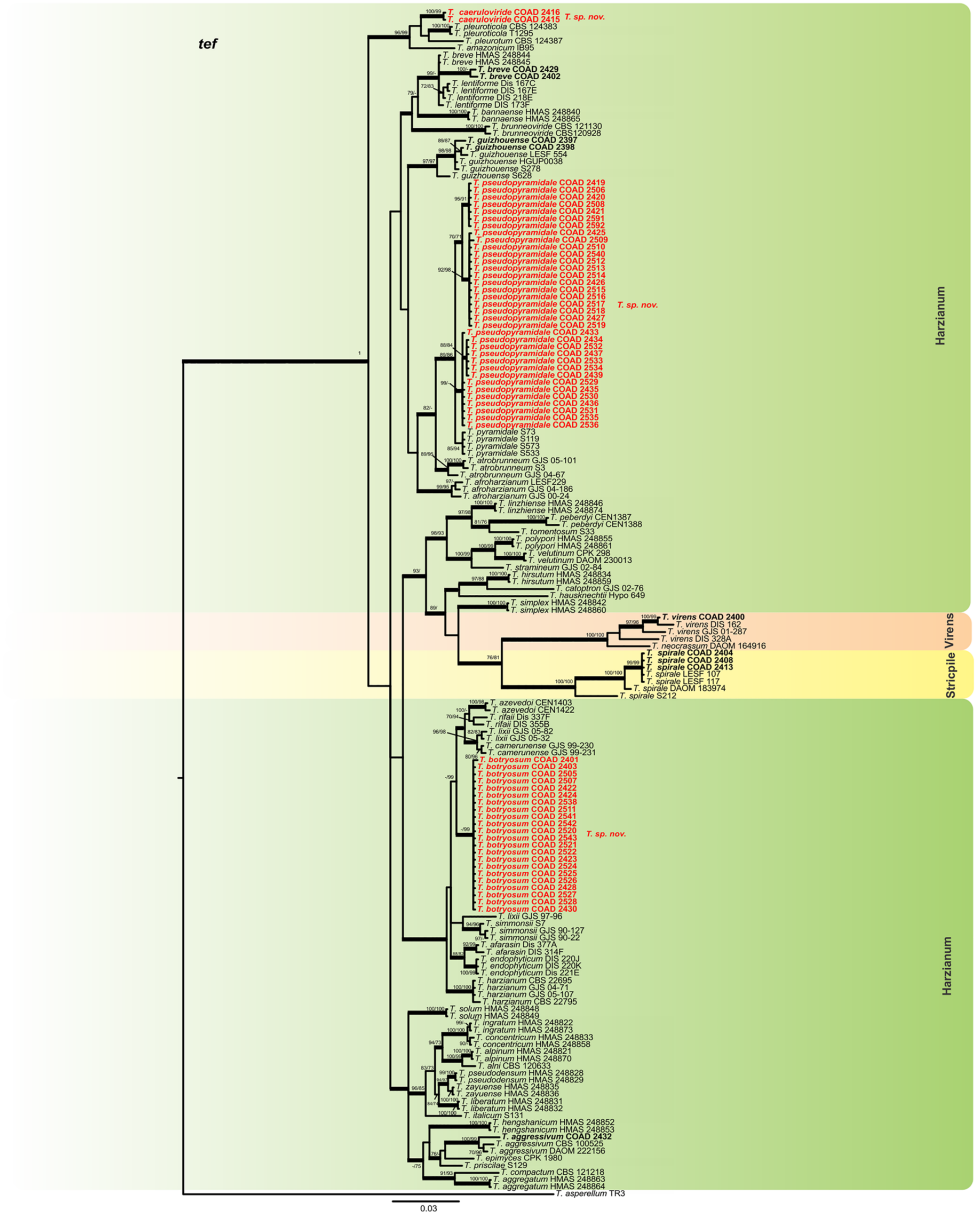
Bayesian phylogenetic tree of clades *Harzianum*, *Strictipile* and *Virens*. The tree was based on tef1 sequence dataset. Bootstrap values (≥ 70%) of the ML analyses, as well as posterior probability scores (≥ 0.9) from a Bayesian analysis of the same dataset, are indicated at well supported nodes together with thickened branches. The isolates belonging to known species, obtained in this study are in bold. Isolates of new species, described in this study, are in bold red. The tree was rooted with *Trichoderma asperellum* (TR3). The phylogenetic tree was edited using Inkscape 1.0 (https://inkscape.org/pt-br/).

**Figure 5:**
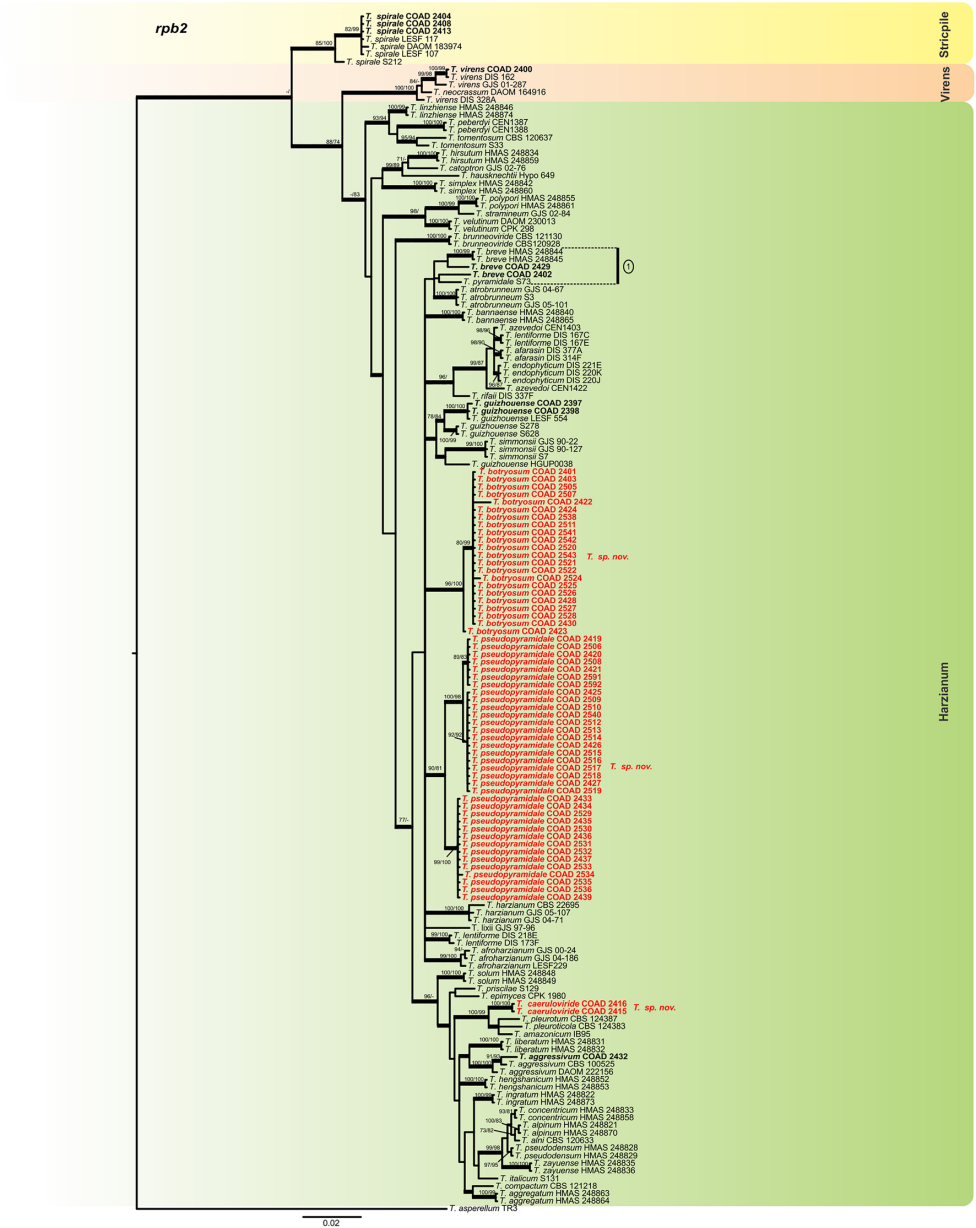
Bayesian phylogenetic tree of clades *Harzianum*, *Strictipile* and *Virens*. The tree was based on rpb2 sequence dataset. Bootstrap values (≥ 70%) of the ML analyses, as well as posterior probability scores (≥ 0.9) from a Bayesian analysis of the same dataset, are indicated at well supported nodes together with thickened branches. The isolates belonging to known species, obtained in this study, are in bold. Isolates of new species, described in this study, are in bold red. The tree was rooted with *Trichoderma asperellum* (TR3). The phylogenetic tree was edited using Inkscape 1.0 (https://inkscape.org/pt-br/).

By following the identification manual for *Trichoderma*^5^, five clades were identified amongst our *Trichoderma* isolates, namely clades: *Viride, Virens, Strictipile, Longibrachiatum* and *Harzianum*. Five isolates were grouped into three known species belonging to the clade *Harzianum*: *T. breve*^12^, *T. guizhouense*^55^ and *T. aggressivum*^56^; one isolate was identified as *T. virens* in the clade *Virens*^57^, and three as *T. spirale* in the clade *Strictipile*^58^ (Fig. 1). Three isolates were grouped in *T. parareesei*^59^, belonging to the *Longibrachiatum* clade and an additional isolate, obtained from Brazil (as a mycoparasite of CLR pustules), also fell within this clade, and was identified as *T. andinense*^2^ (Fig. 2)*.* Twenty-one isolates were grouped into five species of the *Viride* clade: *T. koningiopsis*^60^*, T. petersenii*^60^*, T. theobromicola*^22^*, T. hamatum*^61^ and *T. atroviride*^62^ (Fig. 2). Fifty-nine isolates grouped in three phylogenetic species belonging to the clade *Harzianum* and one isolate belonging to the *Viride* clade did not correspond to any known species and were considered as new taxa, described in this work as: *T. lentissimum* sp. nov., *T. caeruloviride* sp. nov.., *T. botryosum* sp. nov. and *T. pseudopyramidale* sp. nov. (Figs. 1 and 2). In order to clarify the phylogenetic relationship between *T. pseudopyramidale* sp. nov. and *T. pyramidale*, an analysis was performed with the addition of calmodulin sequences. The results of the analysis supported the distinction between *T. pyramidale* and the new species (Fig. 3). The isolates identified in this study as *T. pseudopyramidale* were positioned as paraphyletic with *T. pyramidale* reference isolates, in the *tef* tree (Fig. 4) and in the *rpb2* tree (Fig. 5), the sequence of the single available reference isolate of *T. pyramidale* (S73) was distant from the *T. pseudopyramidale* clade.

**Figure 6:**
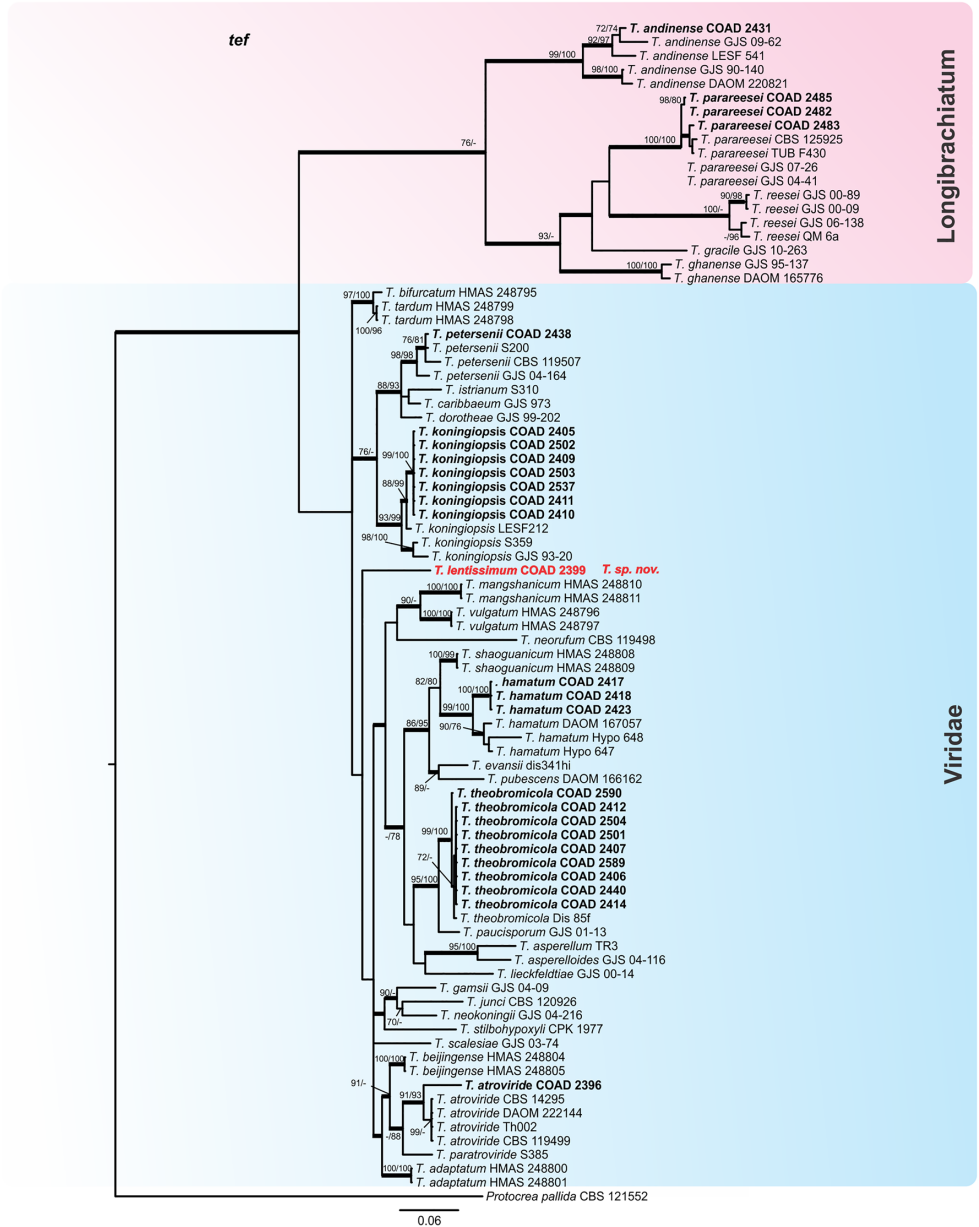
Bayesian phylogenetic tree of clades *Longibrachiatum* and *Viride*. The tree was based on tef1 sequence dataset. Bootstrap values (≥ 70%) of the ML analyses, as well as posterior probability scores (≥ 0.9) from a Bayesian analysis of the same dataset, are indicated at well supported nodes together with thickened branches. The isolates from known species obtained in this study are in bold. Isolates belonging to new species, described in this study, are in bold red. The tree was rooted with *Protocrea pallida* (CBS 121552). The phylogenetic tree was edited using Inkscape 1.0 (https://inkscape.org/pt-br/).

**Figure 7:**
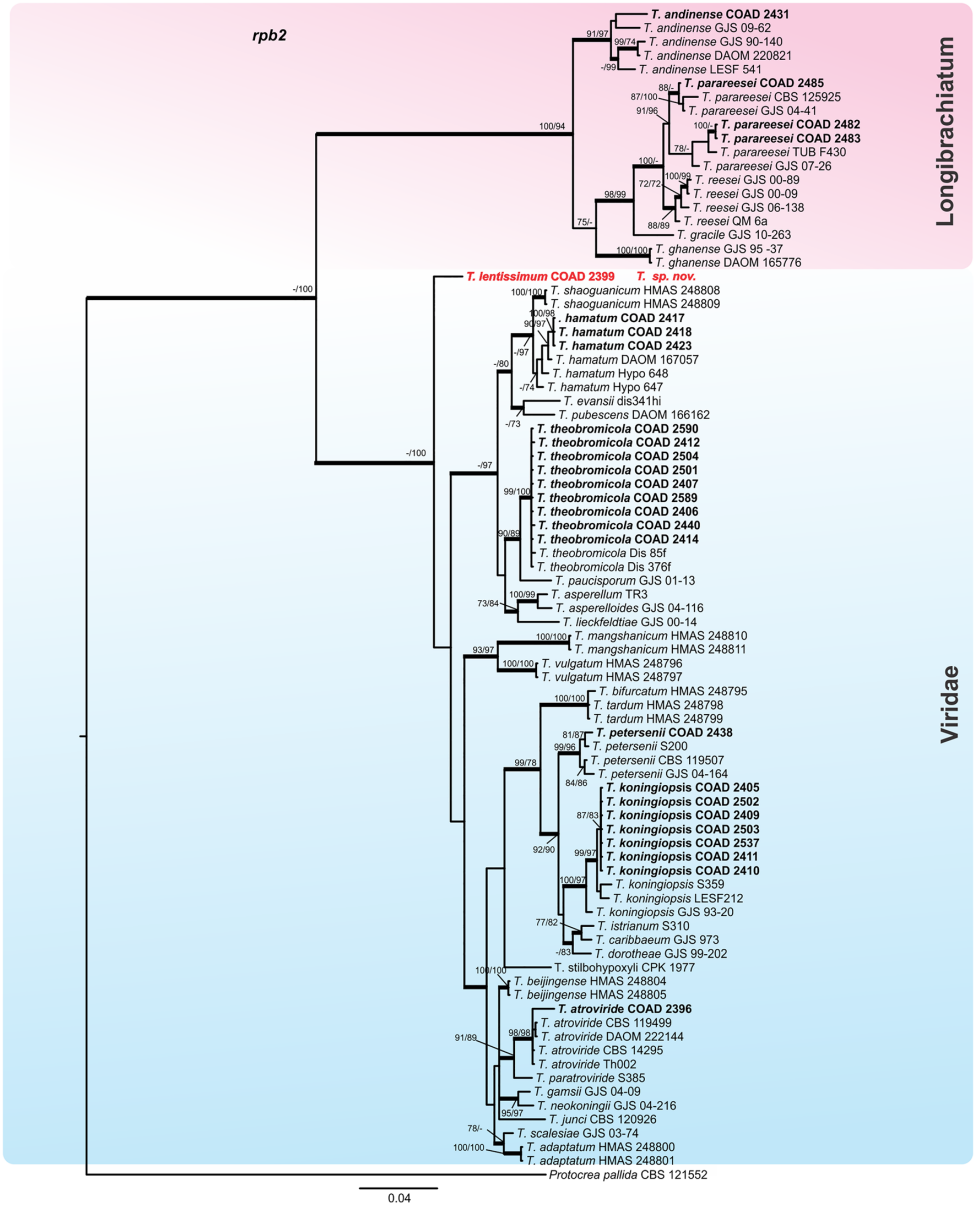
Bayesian phylogenetic tree of clades *Longibrachiatum* and *Viride*. The tree was based on rpb2 sequence dataset. Bootstrap values (≥ 70%) of the ML analyses, as well as posterior probability scores (≥ 0.9) from a Bayesian analysis of the same dataset, are indicated at well supported nodes together with thickened branches. The isolates belonging to known species, obtained in this study are in bold. Isolates of new species, described in thes study, are in bold red. The tree was rooted with *Protocrea pallida* (CBS 121552). The phylogenetic tree was edited using Inkscape 1.0 (https://inkscape.org/pt-br/).

The single *tef* and *rpb2* trees for the *Longibrachiatum* and *Viride* clades were highly congruent (Figs. 6 and 7) with the topology of the concatenated tree (Fig. 2); the same was observed with the individual trees *tef* and *rpb2* of the clade *Harzianum* (Figs. 4 and 5) with the topology concatenated (Fig. 1), except for the isolates identified as *T. breve* (indicated with number 1, Fig. 5), when evaluated in the *rpb2* tree. The reference isolates of this species were placed in two polyphyletic species and one of the isolates of this study attributed to *T. breve* were positioned outside the monophyletic groups (they remained as singletons) in this analysis. In the *tef* tree the clades *Stricpile* and *Virens* divided the largest clade (*Harzianum*) (Fig. 4), however the grouping of the species identified in this study were similar to the concatenated tree (Fig. 1).

### Diversity and distribution

Although the collections of plant material were not systematic—and, therefore, there was no purpose in quantifying the frequency of colonization of plants by species of *Trichoderma* in this study—it was possible to identify patterns of occurrence of taxa, in terms of region/locality, host *Coffea* species and plant tissue type. For example, it was observed that *T. koningiopsis* was isolated only from leaves; whilst *T. theobromicola, T*. *guizhouense* and *T. spirale* were isolated exclusively from stems; and *T. caeruloviride* sp. nov. was isolated from berries only*.* The other species were distributed in more than one plant tissue type. The predominant taxon in all plant tissues was *T. botryosum* sp. nov., but this species was never found as a mycoparasite, whereas *T. pseudopyramidale* sp. nov. was isolated both as an endophyte and as a mycoparasite—almost exclusively from Ethiopia—being common on *Hemileia* rust in forest coffee populations. The species *T. aggressivum, T. andinensis, T. parareesei* and *T. petersenii*, were isolated only once during the survey, from Kenya, Brazil, and Ethiopia, respectively. In Cameroon, 24 isolates belonging to seven species were found, namely: six species in stem samples (*T. botryosum* sp. nov., *T. koningiopsis, T. breve, T. spirale, T. theobromicola* and *T. virens*), one in leaves (*T. koningiopsis*), with one isolated only as a mycoparasite (*T. pseudopyramidale* sp. nov.) (Table 2).

**Table 2:** Number of taxa collected in this survey per country-source.

Species	Ethiopia				Cameroon				Kenya			
	Leaf	Stem	Berry	Mycoparasite	Leaf	Stem	Berry	Mycoparasite	Leaf	Stem	Berry	Mycoparasite
*T. aggressivum*												1
*T. atroviride*									1			
*T. botryosum* sp. nov.	6	11	3			2						
*T. caeruloviride* sp. nov.			2									
*T. guizhouense*										2		
*T. hamatum*		2	1									
*T. koningiopsis*					6	1						
*T. breve*			1			1						
*T. lentissimum* sp. nov.										1		
*T. parareesei*		2		1								
*T. petersenii*				1								

In Ethiopia, 64 isolates belonging to seven species were isolated, namely: 20 isolates of *T. botryosum* sp. nov. from leaves, stems and berries; 34 isolates of *T. pseudopyramidale* sp. nov., from leaves and stems, and as mycoparasites; three isolates of *T. parareesei* from stems and one as a mycoparasite; three isolates of *T. hamatum* from stems and berries; two isolates of *T. caeruloviride* sp. nov. from berries; one isolate of *T. petersenii* growing as a mycoparasite and one isolate of *T. breve* from berries (Table 2). In Kenya, four species were collected, namely: one isolate of *T. aggressivum* growing as a mycoparasite*;* one isolate of *T. atroviride* from leaves; one isolate of *T. guizhouense* and one of *T. lentissimum* sp. nov. from stems (Table 2). When the diversity of *Trichoderma* from coffee in West Africa (Cameroon) is compared with that of East Africa (Kenya and Ethiopia), *T. breve*, *T. pseudopyramidale* sp. nov. and *T. botryosum* sp. nov. are the only species which are present both in Ethiopia and Cameroon. The four species found in Kenya were absent from coffee samples in the other countries. Although this suggests strong endemism and isolation of the *Trichoderma* mycobiota of coffee in Kenya, this should be treated with caution since sampling in Kenya was limited and, thus, this may be an artefact. However, the fact that there was no commonality in species between Kenya and the other countries is intriguing and warrants further investigation. The only Brazilian isolate was identified as *T. andinense*. The occurrence of *T. andinense* appeared in an ad hoc isolation during a search for mycoparasites of CLR pustules in Brazil.

The coffee survey in Brazil—aimed at obtaining endophytic *Trichoderma* from plants growing in semi-wild conditions at eight localities in four states (Table 3)—yielded isolates comprising a range of different genera but just a single isolate was identified as belonging to the genus *Trichoderma*.

**Table 3:** Survey sites in Brazil of *Coffea arabica*, in semi-wild or forest situations, sampled for presence of *Trichoderma* endophytes.

State	Municipality	Situation/locality	Date
Espírito Santo	Domingos Martins	Inside forest fragment, Pedra Azul, Fazenda Camocim	24 Oct 2019
Espírito Santo	Domingos Martins	Inside forest fragment, INCAPER	24 Oct 2019
Espírito Santo	Venda Nova do Imigrante	Inside forest reserve, Fazenda Soloença	24 Oct 2019
Minas Gerais	Cláudio	Surviving plants of abandoned coffee plantation overgrown by secondary forest, vicinity of Cládio city	10 Feb 2019
Minas Gerais	São Miguel do Anta	Insided forest reserve in coffee farm,	9 Oct 2019
Minas Gerais	Viçosa	Inside forest reserve, Mata do Paraíso, Universidade Federal de Viçosa	19 Feb 2019
Rio de Janeiro	Rio de Janeiro	Under secondary forest, outskirts of the Parque Nacional da Floresta da Tijuca	2 Apr 2019
São Paulo	Iporanga	Row of old plants cultivated close to forest reserve (PETAR), Bairro Serra de Iporanga	17 Feb 2020

When comparing the number of taxa of *Trichoderma* present in *C. canephora* and *C. arabica*, it was found that both harbour five species but these are distributed differently in the host tissues. The only *Trichoderma* spp. occurring in both *C. arabica* and *C. canephora* were *T. breve* and *T. botryosum* sp. nov. In *C. arabica*, the highest diversity of *Trichoderma* was found in berries (four species), whereas in *C. canephora* the highest diversity was found in stems (four species): however, since most of the sampling was focused on *C*. *arabica*, it would be premature to reach a firm conclusion. Stems yielded the highest number of isolates from the aerial tissues of *Coffea*, with 40 (42.6% of the total), followed by leaves with 29 (30.8%), and seven isolates (7.4%) from berries. Mycoparasites, with 18 isolates, formed 19.2% of the total (Tables 1 and 2).

### Taxonomy

Four additions to the genus *Trichoderma* emerged from the phylogenetic study of the isolates obtained during this survey of *Coffea* in Africa. Morphological and cultural information proved useful to confirm their separation from closely-related known species of *Trichoderma*; providing further evidence for their recognition as novel species. Type cultures were deposited in the internationally-recognized culture collection of the Universidade Federal de Viçosa (COAD). The following species were collected and identified, with authority names and publication details as recommended by Bissett et al*.*^52^.

***Trichoderma aggressivum*** Samuels & W. Gams—in Samuels et al*., Mycologia* **94**: 167, 2002. MycoBank: MB484638.

Description and illustration, see^5,56^.

*Material examined*. KENYA: Eastern Province, Marsabit National Park, Lake Paradise, primary forest, alt 1340 m: isolated as a mycoparasite of *Hemileia vastatrix* on leaves of *Coffea* cf. *arabica*, H.C. Evans & R.W. Barreto (culture COAD 2432).

Notes: *Trichoderma aggressivum*, in the *Harzianum* clade, has been reported to cause the green mould epidemic in commercially grown *Agaricus bisporus* in North America^61^. This is the first report of *T. aggressivum* as a mycoparasite of *Hemileia* and of rusts, in general; having been recorded previously only in mushroom farms in both North America and Europe^5^. Thus, this appears to be the first record of *T. aggressivum* from the tropics. It has been shown to produce various antifungal compounds^63^, and falls within the clade defined by a mycoparasitic mode of nutrition in a consensus phylogenetic tree^24^.

***Trichoderma andinense*** (Samuels & O. Petrini) Samuels, Jaklitsch & Voglmayr—in Jaklitsch & Voglmayr, *Mycotaxon* **126**:146, 2014. MycoBank: MB807417.

Synonym: *Hypocrea andinensis* Samuels & O. Petrini—in Samuels et al*.*, *Stud. Mycol*. **41**:13, 1998.

Description and illustration, see^2,64^.

*Material examined*. BRAZIL: Rio de Janeiro, Duas Barras, Fazenda do Campo, Sítio Recanto do Sossego, coffee farm, isolated as a mycoparasite of *Hemileia vastatrix* on leaves of *Coffea arabica*, 2 May 2015, R. W. Barreto (culture COAD 2431).

Notes: This fungus, in the *Longibrachiatum* clade, was originally described from its sexual morph collected from a log in the Venezuelan Andes^65^. Similar isolates from soil in Saudi Arabia, Amazonian Peru and Hawaii were reported later^64^ but were considered to represent new taxa within the *T. andinense* sub-clade and this species “remains known only from a single collection”^64^. Thus, this is the first report of *T. andinense* as a mycoparasite of *H. vastatrix*, and as a mycoparasite, in general. It was shown to group in a plant saprotrophy clade in the study by Chaverri and Samuels^24^.

***Trichoderma atroviride*** P. Karst.—in *Finl. Mögelsvamp.* **21**, 1892. MycoBank: MB451289.

Synonym: *Hypocrea atroviridis* Dodd, Lieckfeldt & Samuels, *Mycologia* **95**:36, 2003.

Description and illustration, see^5^.

*Material examined*. KENYA: Western Region, Nandi County, Kakamega Forest Reserve, Isecheno, coffee farm, alt 1600 m; isolated as an endophyte from leaf *of Coffea arabic*a, 23 January 2015, H*.*C*.* Evans & R.W. Barreto (culture COAD 2396).

Notes: This is a cosmopolitan species, in the *Viride* clade, but it is more commonly isolated from soil in tropical regions^5^ and its sexual morph is rarely formed^3^. Chaverri and Samuels^24^ show its habitat preference is soil, but in their phylogenetic tree, *T. atroviride* groups with species having a mycoparasitic mode of nutrition. In our study, it was isolated from a leaf of *C*. *arabica* and this is the first record of *T*. *atroviride* as an endophyte of coffee. *Trichoderma atroviride* is more associated with the plant rhizosphere, rather than aerial tissues, and it has been shown recently that colonization of both maize and tomato roots by this fungus induces foliar herbivory resistance^66^. Previously, it has been known to protect plants against root pathogens (*Pythium*, *Rhizoctonia*) through induced resistance and antibiosis^67^.

**Figure 8:**
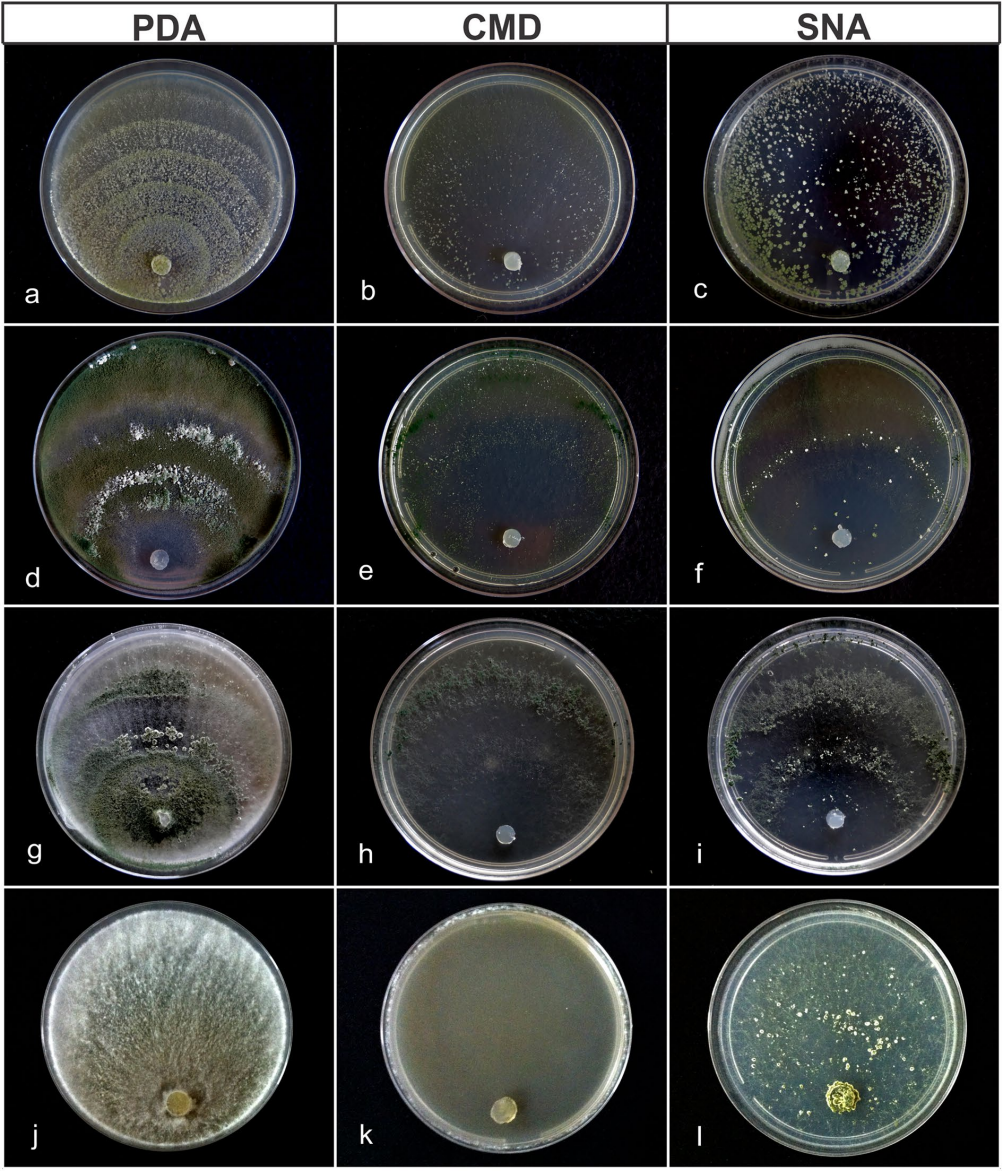
Colony characteristics of the new *Trichoderma* species on PDA, CMD and SNA. All colonies incubated at 25 °C under a 12 h day/night light regime and photographed on day seven. **(a–c**) *T*. *lentissimum* sp. nov..; (**d–f**) *T*. *caeruloviride* sp. nov.; (**g–i**) *T*. *botryosum* sp. nov.; (**j–l**) *T*. *pseudopyramidale* sp. nov.

**Figure 9:**
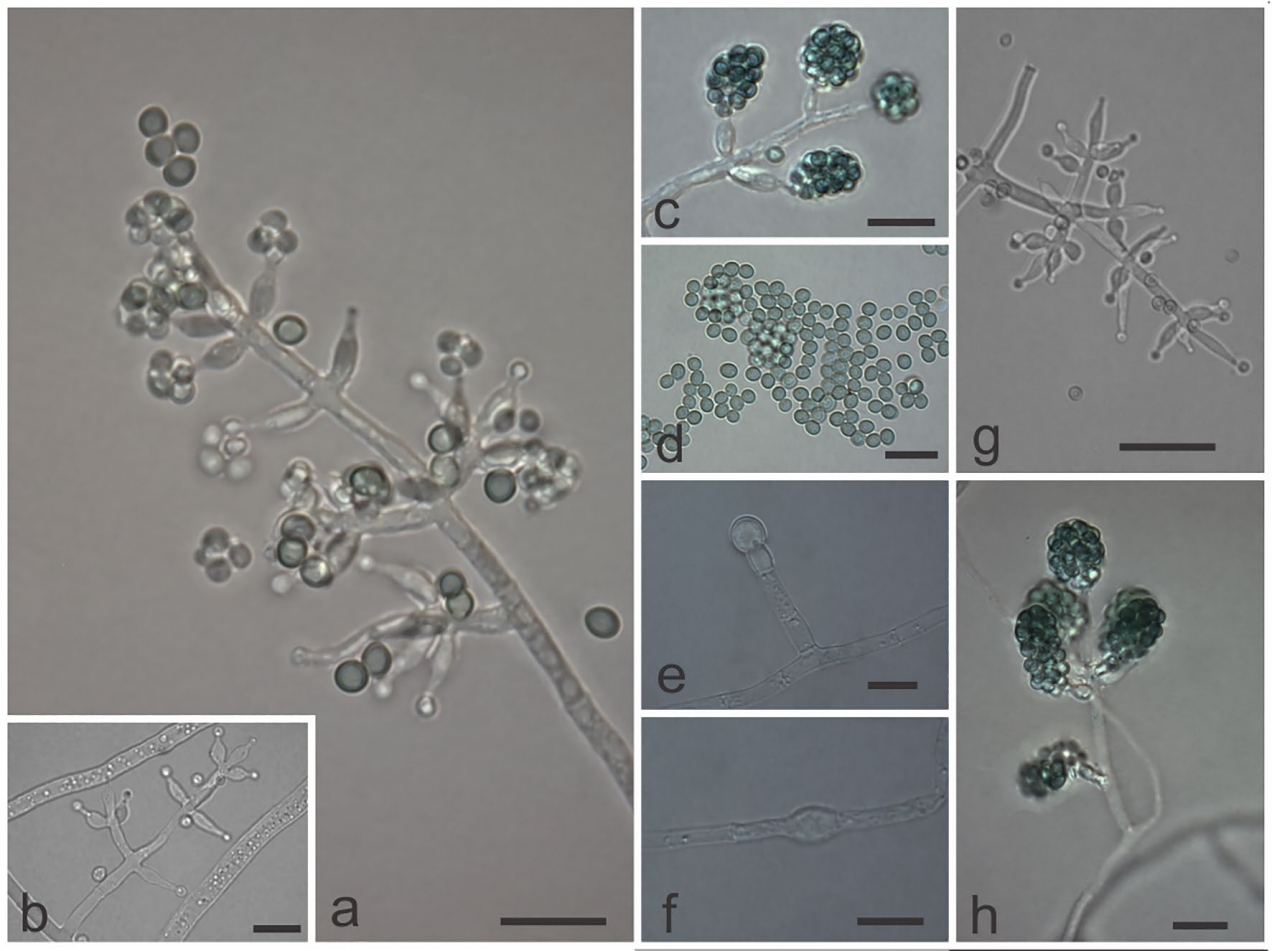
Morphological features characteristic of *Trichoderma botryosum* sp. nov. (COAD 2422). (**a**, **b**, **g**) Conidiophores and phialides formed on SNA. (**c**, **h**) Conidia grouped in bunches. (**e**, **f**) Chlamydospores on CMD. (**d**) Conidia. Bars: (**a**–**f**, **h**) = 10 µm; (**g**) = 20 µm.

***Trichoderma botryosum ***M.C.H. Rodríguez, H.C. Evans & R.W. Barreto **sp. nov.**—MycoBank: MB832341 (Figs. 8g–i, 9).

*Etymology*: referring to the grape-like clusters of conidia.

*Holotype.* ETHIOPIA: Southern Nations, Nationalities and Peoples Region, Kaffa Zone, Bonga District, Gela Wild Coffee Biosphere Reserve, cloud forest, alt 1900 m; isolated as an endophyte from berries of *Coffea arabica*; 25 November 2015, H.C. Evans, K. Belachew & R.W. Barreto. Ex-type culture: COAD 2422. GenBank: *TEF1* = MK044119; RPB2 = MK044212.

Culture characteristics—Colonies on PDA: Optimum growth temperature at 30 °C, 65 mm after 72 h, 56–61 mm at 25 °C, 25 mm at 35 °C; covering the plate after 96 h; cottony white aerial mycelium, green sporulation starting from the centre of the plate, with the formation of concentric rings; with a sweet odour but absence of exudates and soluble pigments. Colonies on CMD: Optimum growth temperature at 30 °C; 63 mm after 72 h, 55–58 mm at 25 °C, 43 mm at 35 °C; mycelium hyaline, poor sporulation, concentric rings absent and no odour. Colonies on SNA: Optimum growth temperature at 30 °C, 61 mm after 72 h, 49–53 mm at 25 °C, 28 mm at 35 °C; mycelium hyaline and smooth, green sporulation; forming concentric rings; exudates and soluble pigments absent.

Conidiophores pyramidal; phialides in whorls or pairs, lateral or terminal, lageniform to ampulliform, 4.0–8.0 (−8.6) × (1.9–) 2.3–3.1 µm (L/W), 1.2–2.4 µm in width at the base; supporting cells 5.4–15.6 × 1.8–3.4 µm (L/W); conidia globose to broadly ovoid, 1.4–3.3 × 1.6–2.8 µm (L/W), green, smooth; chlamydospores abundant, globose to ellipsoidal, terminal and intercalary, 4.4–8.1 × 3.8–7.3 µm (L/W).

Notes: *Trichoderma botryosum* grouped phylogenetically close to *T. afarasin* and *T*. *endophyticum*^4^ in the *Harzianum* clade. The new species is morphologically similar to its close relatives in: the pyramidal-type conidiophores; size of conidia and ampulliform phialides; and growth rate on PDA at 25 °C. The most distinctive morphological features in this species are the presence of chlamydospores and the grape-like clusters of conidia. This appears to be a common endophyte of wild *C*. *arabica* in Ethiopia; being isolated from all the aerial tissues, with 20 isolates recorded (Table 2). It was uncommon in Cameroon with only two isolates from coffee stems.

***Trichoderma breve*** K. Chen & W.Y. Zhuang—Chen & Zhuang., *Sci. Rep.* **7 (no. 9090)**: 7, 2017. MycoBank: MB809992.

Description and illustration, see^12^.

*Material examined*. CAMEROON: South-West Province, Busumbo, Mt. Etinde, coffee farm, 450 m; isolated as endophyte from stem of *Coffea canephora*, 17 November 2015, H.C. Evans, R.W. Barreto & M. K. Ndacnou (culture COAD 2402). ETHIOPIA: Southern Nations, Nationalities and Peoples Region, Kaffa Zone, Bonga District, Komba Wild Forest Reserve, cloud forest, 2000 m; isolated as an endophyte from a berry of *Coffea arabica*, 25 November 2015, H.C. Evans, R.W. Barreto & K. Belachew (culture COAD 2429).

Notes: *Trichoderma breve* is similar morphologically to the *T. harzianum* complex, and has previously been isolated from soil in the north of China^12^. Phylogenetic analyses indicate that *T. breve* is closely related to *T. bannaense*^12^, and in our study it lies close to *T. lentiforme*. This is the first report of *T. breve* in Africa and also as an endophyte of coffee; being recorded from both *C. canephora* and *C. arabica* in Cameroon and Ethiopia, respectively, with a single isolate from each country (Table 2).

**Figure 10:**
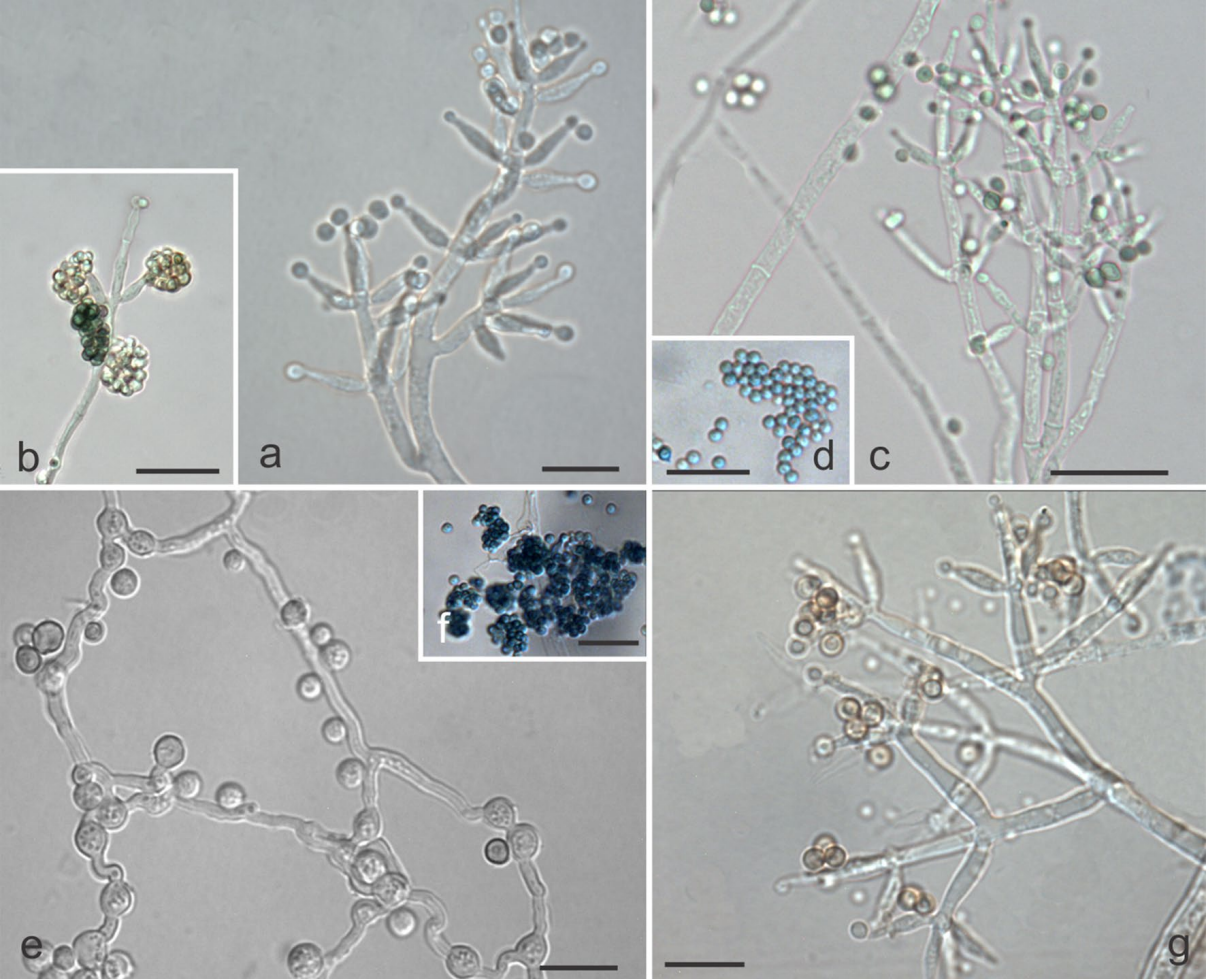
Morphological features characteristic of *Trichoderma caeruloviride* sp. nov. (COAD 2416). (**a**, **b**, **c**, **g**) Conidiophores and phialides formed on SNA and CMD. **e** chlamydospores on CMD. (**f**, **d**) Blue conidia. Bars: (**a**, **e**, **g**) = 10 µm; (**b**–**d**, **f**) = 20 µm.

***Trichoderma caeruloviride*** M.C.H. Rodríguez, H.C. Evans & R.W. Barreto, **sp. nov.** –Mycobank: MB832340 (Figs. 8d–f, 10).

*Etymology*: referring to the blue-green colour of the conidial mass.

*Holotype*: ETHIOPIA: Southern Nations, Nationalities and Peoples Region, Kaffa Zone, Bonga District, Gedam Village, coffee farm, alt 1550 m; isolated as an endophyte from berries of *Coffea arabica*. 25 November 2015, H.C. Evans, K. Belachew & R.W. Barreto. Ex-type culture: COAD 2415. GenBank: *TEF1* = MK044109; RPB2 = MK044202.

Culture characteristics—Colonies on PDA: Optimum growth at 25 °C, 51–53 mm after 72 h, 34 mm at 30 °C, no growth at 35 °C, covering the plate after 96 h; mycelium white, aerial, low with green sporulation, no formation of concentric rings; lacking pigmentation and odour. Colonies on CMD: Optimum growth temperature at 25 °C, 42–44 mm after 72 h, 19 mm at 30 °C, no growth at 35 °C; mycelium hyaline, poor sporulation, green conidia, no concentric rings present and no odour. Colonies on SNA: Optimum growth temperature at 25 °C, 29–36 mm after 72 h, 12 mm at 30 °C, no growth at 35 °C; mycelium hyaline and smooth, poor sporulation; green to blue conidia, with the formation of concentric rings; no pigmentation in the medium.

Conidiophores pyramidal with verticillate, paired lateral branches; phialides generally formed on terminal branches, in divergent whorls of three to four, (5.2–) 5.3–12.2 (–13.2) × (1.7–) 2.0–2.8 (–3.48) µm, mean 7.4 × 2.5 µm (L/W); supporting cells (5.4–) 7.9–9.7 (–10.1) × 1.7–2.0 (–3.0) µm, mean 8.5 × 1.9 µm (L/W); conidia ellipsoidal to ovoid, green, smooth, 2.2–3.0 (–3.2) × (1.9–) 2.3–3.1 (–3.4) µm, mean 2.8 × 2.8 µm (L/W); chlamydospores terminal and intercalary, globose, 3.3–5.0 (–6.6) × 3.0–4.6 (–5.3) µm, mean 4.4 × 3.7 µm (abundantly formed on CMD after 4 days).

Notes: Phylogenetic analyses placed *T. caeruloviride* close to *T. amazonicum*^8^ and *T. pleuroticola*^68^ in the *Harzianum* clade. The new species can be distinguished from its nearest relatives by: no growth at 35 °C; the presence of a coconut-like odour on PDA; and the blue-green conidia in SNA microculture. Morphologically, *T. caeruloviride* is distinct from *T. amazonicum* which has a branching pattern of the pachybasium type, elliptical to subglobose conidia, ampulliform phialides, and chlamydospore-like structures in clusters. *Trichoderma caeruloviride* shares some morphological characteristics with *T*. *pleuroticola*, such as the pyramidal-type branching pattern, the globose conidia and the formation of chlamydospores; but can be separated by the larger, lageniform phialides of *T. caeruloviride*. Both isolates were from berries of *C*. *arabica* in Ethiopia and it appears to be a rare species (Table 2).

***Trichoderma guizhouense*** Q.-R. Li, E.H.C. McKenzie & Yong Wang—in Li et al*.*, *Mycol. Prog*. **12**:170, 2012. MycoBank: MB563664.

Description and illustration, see^4,55^.

*Material examined*. KENYA: Eastern Province, Marsabit National Park. Lake Paradise, primary forest, alt 1340 m; isolated as an endophyte from stem of *Coffea* sp. (wild population close to *C. arabica* and *C. canephora,* but identity uncertain, even after examination by an authority on the genus *Coffea*) H.C. Evans & R.W. Barreto, 28 January 2015 (cultures COAD 2397 and COAD 2398).

Notes: *Trichoderma guizhouense*, in the *Harzianum* clade, was first isolated from soil in Guizhou Province of China^55^, and has since shown promise as a biocontrol agent of *Rhizoctonia* root rot^69^. It was also isolated as an endophyte from the woody liana, *Ancistrocladus korupensis* (Ancistrocladaceae)—extracts of which are active against HIV^70^—and from the sapwood of *Cola* spp. (Malvaceae) in rainforest of the Cameroon Republic^4^ (H.C. Evans unpubl.). This is the first report of T. *guizhouense* as a coffee endophyte.

***Trichoderma hamatum*** (Bonord.) Bainier—*Bull. Soc. Mycol. Fr.* **22**:131, 1906. MycoBank: MB165799.

Synonyms: *Verticillium hamatum* Bonord., *Handb. Allgem. Mykol*.: 97, 1851. *Pachybasium hamatum* (Bonord.) Sacc., *Rev. Mycol. Toulouse* **7**:161, 1885. *Phymatotrichum hamatum* (Bonord.) Oudem., *Ned. Kruidk. Archf.* **3**:908, 1903.

Description and illustration, see^3,5^.

*Material examined*. ETHIOPIA: Southern Nations, Nationalities and Peoples Region, Kaffa Zone, Bonga District, Gedam Village, coffee farm, alt 1550 m, isolated as an endophye from stems of *Coffea arabica*, 25 November 2015, H.C. Evans, R.W. Barreto & K. Belachew (cultures COAD 2418 and COAD 2417). ETHIOPIA: Southern Nations, Nationalities and Peoples Region, Kaffa Zone, Bonga District, Gela Wild Coffee Biosphere Reserve, cloud forest, alt 1700 m; isolated as an endophyte from berry of *Coffea arabica*, 25 November 2015, H.C. Evans, R.W. Barreto & K. Belachew (COAD 2423).

Notes: *Trichoderma hamatum*, in the *Viride* clade, is a cosmopolitan species, originally isolated from soil but it has also been reported as an endophyte in both stems and pods of wild *Theobroma gileri* from sub-montane forest in western Ecuador^20^. In addition, it was identified as a mycoparasite of frosty pod disease (*Moniliophthora roreri*) on the same host in this ecosystem^20^. It has also been isolated from the rhizosphere of *C*. *arabica* in Ethiopia^15^. In our study, *T*. *hamatum* was obtained from stems and berries of *C*. *arabica* in both cultivated and wild coffee plants, also in Ethiopia. It is most frequently cited as a colonizer of the rhizosphere, and some soil strains have been shown to promote crop growth, to activate biocontrol mechanisms against root pathogens and to induce systemic resistance to foliar pathogens^71^. Previously, an endophyte strain from the pod of a wild *Theobroma* species was found to promote the growth and delay drought symptoms in cacao plants^72^.

***Trichoderma koningiopsis*** Samuels, C. Suárez & H.C. Evans—in Samuels et al*.*, *Stud*. *Mycol.* **56**:117, 2006. MycoBank: MB487454.

Description and illustration, see^5,60^.

*Material examined*. CAMEROON: Eastern Province, Zemele Village, coffee farm, alt 660 m; isolated as an endophyte from leaves of *Coffea canephora*, 22 November 2015, H.C. Evans, R.W. Barreto & M. K. Ndacnou (Cultures COAD 2537; COAD 2405; COAD 2502; COAD 2503; COAD 2410; COAD 2411 and COAD 2409).

Notes: *Trichoderma koningiopsis*, in the *Viride* clade, is a cosmopolitan species, but it is more frequently recorded in tropical rather than temperate regions, and mostly from soil. During a survey of *Trichoderma* diversity in soil and leaf litter from the Amazonian rainforest of Colombia, *T*. *koningiopsis* was amongst the commonest species isolated^73^. In the Atlantic rainforest of Brazil, *T*. *koningiopsis* was found to be the dominant *Trichoderma* species in leaves being carried by *Atta* leaf-cutting ants, and subsequently rejected by them from the nest: it was posited that the ants recognized the threat posed by this mycoparasite to the fungal garden^74^. It has also been reported as a common stem endophyte in a species of *Theobroma* in sub-montane forest in western Ecuador^22^. It has also been shown to colonize cacao plants via the leaf trichomes^75^. An isolate of *Trichoderma koningiopsis* from the stem of a *Vinca* species in Iran was found to produce a range of anti-microbials, including trichodermin, as well as cytotoxic compounds^76^. *Trichoderma koningiopsis* has also previously been isolated from the rhizosphere of *C*. *arabica* in Ethiopia^15^. Here, it is reported for the first time as an endophyte of *Coffea*: all seven isolates being recovered from the leaves and stem of both cultivated and wild *C. canephora* in Cameroon (Table 2), where it appears to be common.

**Figure 11:**
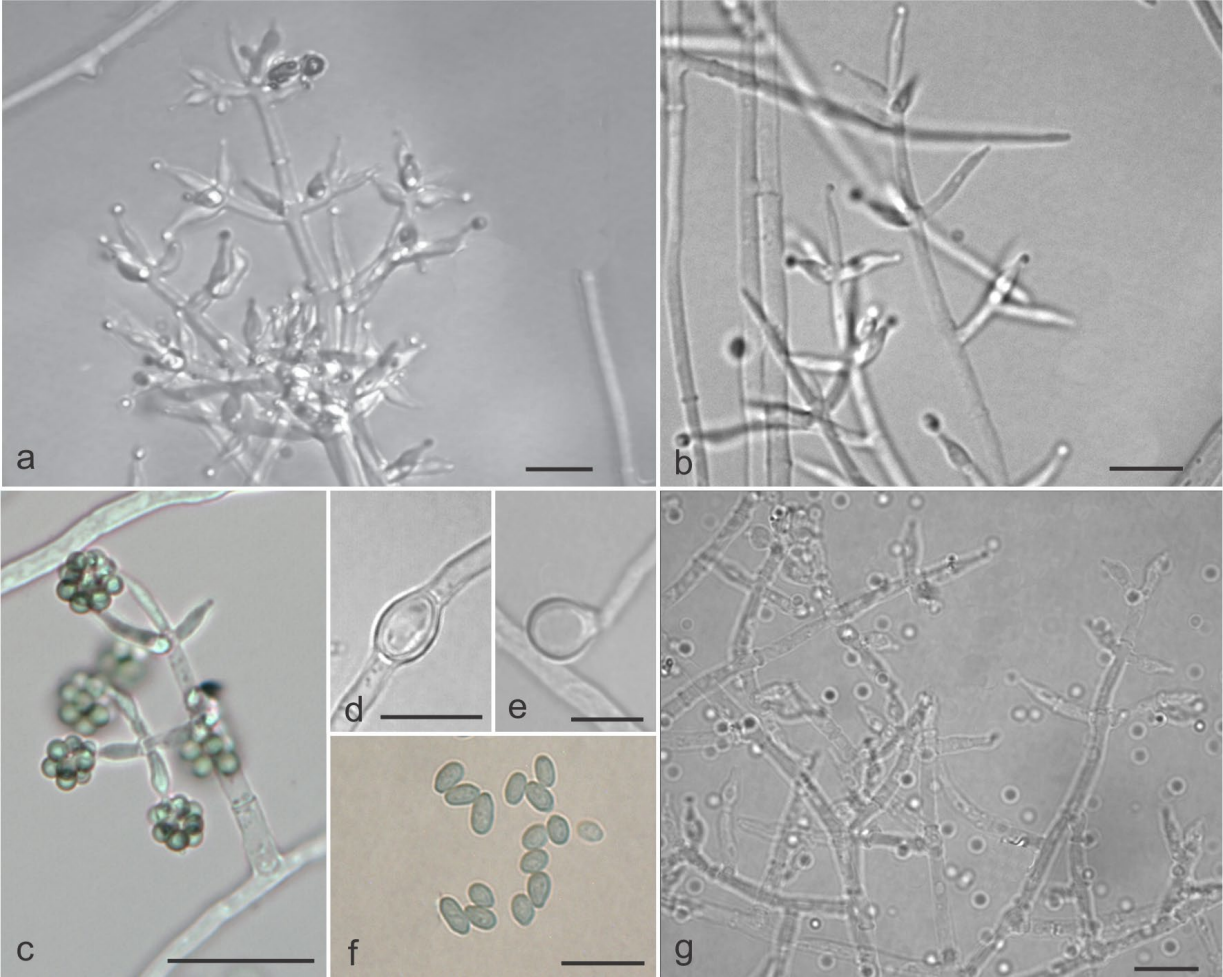
Morphological features characteristic of *Trichoderma lentissimum* sp. nov. (COAD 2399). (**a**–**c**, **g**) Conidiophores and phialides formed on SNA and CMD. (**d**, **e**) Chlamydospores on CMD. (**f**) Conidia. Bars: (**a**, **b**, **d**–**f**) = 10 µm; (**c**, **g**) = 20 µm.

***Trichoderma lentissimum*** M.C.H. Rodríguez, H.C. Evans & R.W. Barreto **sp. nov.** Mycobank: MB832339 (Figs. 8a–c, 11).

*Etymology*: referring to its slow growth in culture.

*Holotype*: KENYA: Eastern Province, Marsabit National Park, Lake Paradise, primary forest, alt 1340 m; isolated as an endophyte from stem of *Coffea* cf. *arabica*, 28 January 2015, H.C. Evans & R.W. Barreto. Ex-type culture: COAD 2399. Genbank: *TEF1* = MK044086; *RPB2* = MK044179.

Culture characteristics—Colonies on PDA: Optimum growth temperature at 25 °C, 28–30 mm after 72 h, 7 mm at 30 °C, no growth at 35 °C; at 25 °C mycelium mostly on surface, greyish-white aerial, olive-green sporulation, beginning in the colony centre with the formation of concentric rings; no pigmentation in the medium; odour lacking. Colonies on CMD: Optimum growth temperature at 25 °C, 29–31 mm after 72 h, 1 mm at 30 °C, no growth at 35 °C; mycelium mainly hyaline, low with olive-green sporulation, no formation of concentric rings, lacking pigmentation and odour. Colonies on SNA: Optimum growth temperature at 25 °C, 30 mm after 72 h, 9 mm at 30 °C, no growth at 35 °C; mycelium hyaline and smooth, low with olive-green sporulation; absence of concentric rings and no pigmentation; forming amorphous and cottony pustules, 1–3.5 mm in diam.

Conidiophores pyramidal with phialides in whorls; phialides lageniform, (4–) 5–9.7 (–10.7) × (1.9–) 2–3 (–3.2) µm, mean 7.6 × 2.7 µm (L/W); supporting cells (4.9–) 5.7–11.9 (–12.5) × (1.6–) 1.7–2.9 (–3) µm, mean 8.1 × 2.1 µm (L/W); conidia globose to broadly ellipsoid, 2.2– 3.9 (–4.3) × (1.9–) 2–2.9 (–3.2) µm, mean 2.8 × 2.6 µm, green-coloured, smooth; chlamydospores globose to subglobose, abundant, intercalary and terminal, (5.4–) 6.9–12.3 (–13.5) × (3.6– 4–7.1 (–10.2) µm, mean 9.4 × 6.6 µm on CMD and PDA.

Notes: *Trichoderma lentissimum* is phylogenetically close to *T. gamsii*^77^ and *T. lieckfeldtiae*^38^ in the *Viride* clade. The new species is also morphologically similar to *T. gamsii* in: the branching pattern (pyramidal type); the lageniform phialides; and the formation of chlamydospores; but can be separated based on the smaller phialides and conidia of *T. lentissimum* as compared with those of *T. gamsii*. The most prominent differences between *T. lentissimum* and *T. lieckfeldtiae,* is the pachybasium-type branching pattern which is found only in *T. lieckfeldtiae* and the absence of chlamydospores in the latter. This species appears to be rare and it was only isolated once during the survey, as a stem endophyte in Kenya.

***Trichoderma parareesei*** Jaklitsch, Druzhin. & Atanasova—in Atanasova et al*. Appl. Environ. Microbiol*. **76**:7261, 2010. MycoBank: MB515503.

Description and illustration, see^59^.

In a paper of the same year (2010)^78^, this species is listed as *T. parareesei nom. prov.* and, subsequently, registered as *T. parareesei* sp. nov.. Jaklitsch, Druzhinina & Atanasova in MycoBank^59^. However, the authorities in MycoBank are cited erroneously as Jacklitsch & Atanasova and, even more bizarrely, as Atan., Jaklitsch, Komoń-Zel., C.P. Kubicek & Druzhin. in Index Fungorum; whilst Bissett et al.^52^ give the authorities as Atanasova, Jaklitsch, Komoń-Zelazowska, C.P. Kubicek & Druzhinina. Moreover, both the latter two quote the wrong citation page, 7259 instead of 7261. The correct citation, as given above, has been confirmed by the editor of Index Fungorum (P.M Kirk pers. comm., 22 January 2020), and the IF database will be amended, accordingly.

*Material examined*. ETHIOPIA: Southern Nations, Nationalities and Peoples Region, Kaffa Zone, Bonga District, Komba Wild Forest Reserve, cloud forest, alt 2000 m; isolated as a mycoparasite of *Hemileia* cf. *coffeicola*, from leaf of *Coffea arabica*, 19 January 2018, H.C. Evans & K. Belachew (culture COAD 2483); *Ibid*, Maakira, coffee farm, alt 1450 m; isolated as an endophyte from stem of *Coffea arabica*, 21 January 2018, H.C. Evans & K. Belachew (culture COAD 2482); Kaffa Zone, Gesha District, coffee farm, alt 1600 m; isolated as an endophyte from stem of *Coffea arabica*, 22 January 2018, H.C. Evans & K. Belachew (culture COAD 2485).

Notes: *Trichoderma parareesei*, in the *Longibrachiatum* clade, was originally isolated from soil of a subtropical rainforest near Iguazu Falls, Argentina, and is reported to have a pantropical distribution in both rainforest and agricultural soils^59,64^. During a survey of *Trichoderma* species in the sapwood and dead branches of cacao trees in south-eastern Brazil, it was exclusively isolated from dead wood—where it was the dominant species—and was never recorded from the sapwood^79^. In our study, *T. parareesei* was isolated as an endophyte from stems of *C*. *arabica* and also as a mycoparasite of *Hemileia* cf. *coffeicola*. This species has never been reported before either as an endophyte in coffee or as a mycoparasite, and probably this is the first record as an endophyte, in general (see^79^). *Trichoderma parareesei* was described as a sympatric, clonal, agamospecies (reproducing only asexually) closely related to *T*. *reesei* and is its likely ancestor^59,78^. The latter is a critically important species in the biotechnology industry as a producer of cellulases and hemicellulases and—because of its ability to express recombinant proteins—it is now being targeted for a role in the production of biofuels^80^. Many of these industrial strains have been shown to be *T*. *parareesei* by Druzhinina et al.^78^. They also found that this species is strongly mycoparasitic, compared to *T*. *reesei*, showing significant antagonism to a range of aerial plant pathogens in dual-culture tests. It has also been demonstrated that *T*. *parareesei* has biocontrol potential against both fungal and oomycete plant pathogens and, moreover, that it enhances root development and promotes growth, in general, of tomato plants^81^. Finally, it was posited that the ecological niche of *T*. *parareesei* is not soil but the canopy of tropical forest^78^. Our results confirm their supposition since this species was recorded in stems of wild to semi-wild *C. arabica*, as well as being found as a mycoparasite of *Hemileia* rust in the canopy of wild coffee trees in the understorey of cloud forest: all isolates from Ethiopia (Tables 1 and 2).

***Trichoderma petersenii*** Samuels, Dodd & Schroers—in Samuels et. al., *Stud. Mycol*. **56**:122, 2006. MycoBank: MB501043.

Synonym: *Hypocrea petersenii* Samuels, Dodd & Schroers. Samuels et al*.*, *Stud. Mycol*. **56**:122, 2006.

Description and illustration, see^60^.

*Material examined*. ETHIOPIA: Southern Nations, Nationalities and Peoples Region, Kaffa Zone, Bonga District, Komba Wild Forest Reserve, cloud forest, alt 1900 m; isolated as a mycoparasite of *Hemileia* cf. *coffeicola* on leaf of *Coffea arabica*, 26 November 2015, H.C. Evans & R.W. Barreto (culture COAD 2438).

Notes: With the exception of the soil isolate DAOM 165782 (North Carolina), *T. petersenii* was known previously only from ascospore isolations^60^. However, it has since shown to be common on woody hosts in Southern Europe, especially in Spain, and is reported to be ubiquitous on the Canary Islands, being found in the sexual stage on stromata of the *Xylariaceae*^3^, presumably as a mycoparasite. In our study, *T. petersenii* was found for the first time in Africa, and also this is the first report of it as a rust mycoparasite, specifically, on a *Hemileia* species close to *H*. *coffeicola* (Table 1). The latter species, however, is a pathogen of *C*. *canephora* in the lowland tropics of West Africa and thus this high-altitude rust of wild Arabica coffee in Ethiopia is considered to be undescribed.

**Figure 12:**
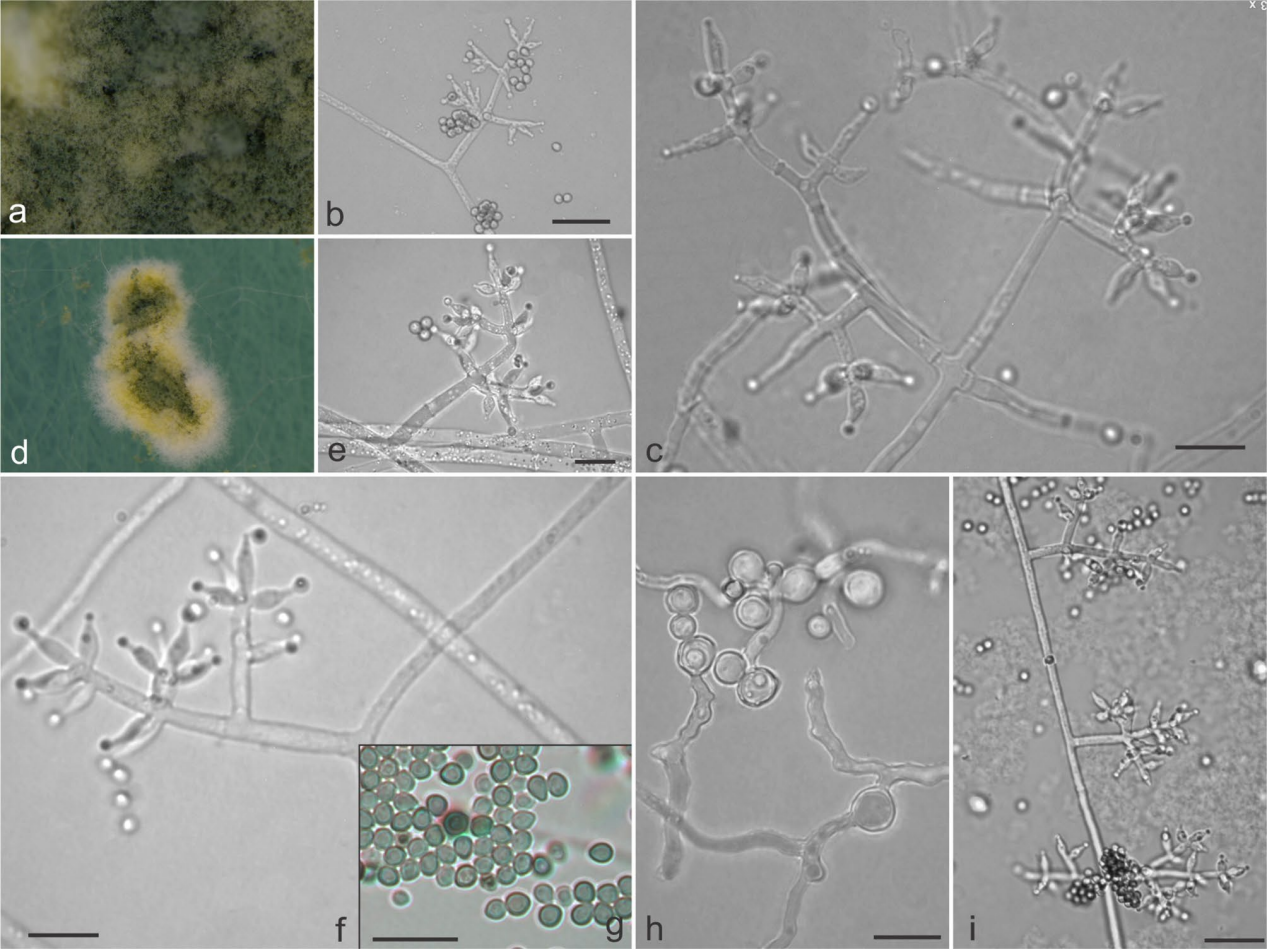
Morphological features characteristic of *Trichoderma pseudopyramidale* sp. nov. (COAD 2426). (**a**, **d**) Stereo microscope images on SNA. (**b**, **c**, **e**, **f**, **i**) Conidiophores and phialides formed on SNA. (**h**) chlamydospores on CMD. (**g**) Conidia. Bars: (**e**, **c**, **f**, **h**) = 10 µm; (**b**, **i**) = 20 µm.

***Trichoderma pseudopyramidale ***M.C.H. Rodríguez, H.C. Evans & R.W. Barreto **sp. nov.**—Mycobank: MB832342 (Figs. 8j–l, 12).

Etymology: indicating its phylogenetic proximity to *T. pyramidale.*

*Holotype*: ETHIOPIA: Southern Nations, Nationalities and Peoples Region, Kaffa Zone, Bonga District, Maakira-Grugutto, semi-wild coffee farm, alt 1600 m; isolated as an endophyte from leaves and stems of *Coffea arabica*, 25 November 2015, H.C. Evans & R.W. Barreto. Ex-type culture: COAD 2426.

Culture characteristics—Colonies on PDA: Optimum growth temperature at 25 °C, 43–45 mm after 72 h, 40 mm at 30 °C, 22 mm at 35 °C, covering the plate after 96 h; mycelium cream, concentric rings absent and no odour; yellow pigmentation in the central reverse of plate. Colonies on CMD: Optimum growth temperature at 25 °C and 30 °C, 43–44 mm after 72 h, 44 mm at 30 °C, 25 mm at 35 °C; mycelium hyaline, no sporulation, absence of concentric rings and odour. Colonies on SNA: Optimum growth temperature at 25 °C, 40–42 mm after 72 h, 33 mm at 30 °C, 23 mm at 35 °C; mycelium hyaline and smooth, sporulation sparse; absence of concentric rings and pigmentation; forming amorphous, cottony pustules, measuring 1–3.5 mm in diam, turning yellowish then dark green centrally.

Conidiophores pyramidal- to tree-type; phialides ampulliform to lageniform, usually formed in whorls, (5–) 5.3–8.6 (–9.1) × (1.9–) 2.2–2.9 (–3.2) μm, mean 6.4 × 2.6 μm (L/W), base (1.3–) 1.3–2.3 (–2.5) µm wide; supporting cells (2.5–) 3.3–7.1 (–8.2) × 1.9–2.8 (–3.2) µm, mean 6 × 2.5 μm (L/W); conidia globose, sub-globose or ovoid, green, smooth, (1.8–) 2.1–2.9 × (2.1–) 2.3–2.9 (–3.2) µm, mean 2.5 × 2.6 μm (L/W); chlamydospores globose to sub-globose (3.21–) 3.2–8.1 (–9.07) × (3.5–) 3.9–7.5 (–8.5) µm, mean 6.0 × 6.1 μm (L/W).

*Additional strain examined*: CAMEROON: Eastern Province, Somalomo Village, coffee farm, alt 700 m; isolated as a mycoparasite from pustules of *Hemileia coffeicola* on leaves of *Coffea canephora*, 22 November 2015, H.C. Evans and R.W. Barreto, COAD 2433. GenBank: *TEF1* = MK044131, MK044157; *RPB2* = MK044224, MK044250; *CAL* = MK084870, MK084869.

Notes: Most of the species found in this study could be identified with high support using the combination of *tef1* and *rpb2* genes. However, for the isolates assigned to *T. pseudopyramidale*, it was also necessary to include the calmodulin gene in the analysis in order to separate the novel species from the closely related *T. pyramidale*. *Trichoderma pseudopyramidale* grouped phylogenetically close to *T. pyramidale*^4^ in the *Harzianum* clade. The two species share several characteristics in common, such as pyramidal conidiophores, a similar growth rate on PDA and SNA, and the formation of amorphous pustules with white-yellow borders. *Trichoderma pyramidale* has larger phialides and conidia, compared to *T. pseudopyramidale*, whilst the latter species forms chlamydospores on CMD and produces a yellow reverse on PDA; and, unlike *T. pyramidale*, it is able to grow at 35 °C. *Trichoderma pseudopyramidale* forms two monophyletic subclades, one containing endophytic isolates and another including isolates obtained directly from CLR pustules (as mycoparasites). Since both subclades come from a phylogenetically well-supported clade by ML, MP and BI, we decided to keep them in a single species and consider them to represent an infra-specific grouping not warranting taxonomic recognition at this stage. No significant differences in morphology or in the growth rates for isolates belonging to these subclades were found. In our study, *T. pseudopyramidale* represented by far the commonest isolate (35 isolates), the greater majority as an endophyte in both stems and leaves of *C*. *arabica*, as well as a mycoparasite of *Hemileia* cf. *coffeicola* on wild Arabica coffee in Ethiopia (Tables 1 and 2). There was a single record from Cameroon, as a mycoparasite of *Hemileia coffeicola* on *C*. *canephora*.

***Trichoderma spirale*** Bissett—*Can. J. Bot.* **69**:2408, 1991. MycoBank: MB359087 Description and illustration, see^5^.

*Material examined*. CAMEROON: Eastern Province, Somalomo, Dja Forest Reserve, rainforest, alt 700 m; isolated as an endophyte from stems of *C*. *canephora*, 21 November 2015, H.C. Evans & R.W. Barreto (cultures COAD 2408; COAD 2404 and COAD 2413).

Notes: *Trichoderma spirale*, in the *Strictipile* clade, is a cosmopolitan species. It was isolated for the first time from soil in Thailand, but has since found to be common in sapwood of cacao and other *Theobroma* spp.^79,82^. It was also the dominant *Trichoderma* species isolated from roots of *Pinus densiflora* in South Korea and it was suggested that *Trichoderma* may be playing a role in stimulating plant colonization by the ectomycorrhizal fungus *Tricholoma matsutake*^83^. In our study, it was found for the first time as an endophyte of coffee; all three isolates were from stems of wild *C. canephora* in Cameroon rainforest of the Congo basin.

***Trichoderma theobromicola*** Samuels & H.C. Evans—in Samuels et al., *Mycol. Res.* **110**:390, 2006. MycoBank: MB356642.

Description and illustration, see^22^.

*Material examined*. CAMEROON: Eastern Province, Somalomo, Dja Forest Reserve, rainforest, 700 m; as an endophyte from stems of *Coffea canephora*, 21 November 2015, H.C. Evans & R.W. Barreto (cultures COAD 2406; COAD 2407; COAD 2501; COAD 2504; COAD 2412; COAD 2440; COAD 2414; COAD 2589 and COAD 2590).

Notes: *Trichoderma theobromicola*, in the *Viride* clade, was found for the first time growing as an endophyte in the trunk of wild cacao in Amazonian Peru and, in subsequent greenhouse studies, it demonstrated promise as an antagonist against frosty pod disease (*Moniliophthora roreri*) after being inoculated into and re-isolated from seedlings of cacao^22^. Similarly, an isolate of *T. theobromicola* from *Cola* sp. in the Cameroon^38^ revealed biocontrol potential after it was shown to be parasitic on and reduced the disease incidence of *Phytophthora* in *Capsicum annuum*^84^. All the isolates in our study were from stems of wild *C*. *canephora* in Cameroon rainforest (Table 1). This is the first report of this species as an endophyte of coffee.

***Trichoderma virens*** (J.H. Hill, Giddens & A.A. Foster) Arx—*Nova Hedwig. Beih.* **87**: 288, 1987. MycoBank: MB128198.

Synonym: *Gliocladium virens* J.H. Mill., Giddens & A.A. Foster, *Mycologia* **49**:792, 1957.

Description and illustration, see^5^.

*Material examined*. CAMEROON: South-West Province, Ekonjo, Mt. Etinde, rainforest, alt 700 m; isolated as an endophyte from stem of wild *Coffea brevipes*, 17 November 2015, H.C. Evans, R.W. Barreto & M.K. Ndacnou (culture COAD 2400).

Notes: *Trichoderma virens* is a cosmopolitan species, commonly isolated from soil samples, but its sexual morph appears to be rare; having been found only once on dead wood^5^. In our study, it was isolated from the stem of a wild species of *Coffea* in Cameroonian rainforest on a single occasion. This is the first record of *T*. *virens* as an endophyte of coffee and, seemingly, as an endophyte of aerial plant tissues. Previously, it has been shown to colonize sugar-cane roots; forming dense mycelium in the intercellular spaces^85^. It was also reported that *T*. *virens* secretes proteins to facilitate colonization of maize roots in which plant-host immune responses are suppressed^86^. Earlier, it was demonstrated that *T*. *virens* promotes growth of *Arabidopsis* by stimulating the root system through an auxin-dependent mechanism^87^. The isolate from our study may have additional mechanisms to colonize woody stems and, perhaps, to form a similar beneficial interaction with its wild coffee host.

## Discussion

Previous studies have investigated the diversity of endophytic fungi associated with coffee^88–92^, but these were based on surveys restricted to the Americas and Hawaii, where coffee is an exotic introduced species. The endophytic mycobiota found in these studies is dominated by genera such as C*olletotrichum, Fusarium, Penicillium, Pestalotia* and *Xylaria*. Such assemblages consist mainly of opportunistic endophytes—seemingly, of little biological significance to their hosts^93^—with *Trichoderma* appearing only infrequently. Only one study involved sampling of all the coffee tissues (leaf, berry, stem, root system)^89^ and, of the 843 isolates obtained, only four were identified as belonging to the genus *Trichoderma*. Conversely, and in sharp contrast, the *Coffea* samples from Africa in this study yielded 76 endophytic isolates of *Trichoderma* from the aerial plant tissues of a relatively small sample size, with a highly diverse taxonomic range, including four new species. At this stage, it is not possible to determine whether the new taxa described herein are geographically restricted to Africa or even to coffee. Nevertheless, we find it significant that a far richer diversity of *Trichoderma* was found in association with coffee in its African centre of origin compared to that elsewhere, especially in the Neotropics. We also find particularly relevant the complete absence of endophitic *Trichoderma* species isolates amongst the plethora of fungal isolates obtained from our sampling in semi-wild situations in Brazil. This was entirely unexpected and may indicate the existence of a ‘*Trichoderma* void’ in the coffee endophyte mycobiota outside of Africa.

The occurrence of *Trichoderma* in association with *C*. *arabica* has been reported previously in Ethiopia^15,94^, but these studies focused on strains isolated from the rhizosphere and root tissues. The isolates included: *T. harzianum *sensu lato, *T. hamatum*, *T. asperelloides*, *T. spirale*, *T. atroviride*, *T. koningiopsis*, *T. gamsii* and *T. longibrachiatum.* Only three of these taxa were isolated during our study of stems, leaves and berries: namely, *T. hamatum*, *T. spirale* and *T. koningiopsis.* These are cosmopolitan species that are frequently isolated from tropical habitats, especially from soil^6,7,60,95^. Certain *Trichoderma* species were isolated from more than one plant tissue type: *T. koningiopsis* and *T. spirale* from the leaves and stems of *C. canephora*; *T. hamatum* from the stems and berries of *C. arabica*. *T. hamatum, T. koningiopsis* and *T. spirale* have also been reported as endophytes in other tropical woody plants, notably cacao and rubber^5^. Nevertheless, only *T. hamatum* had previously been reported as endophytic in *C. arabica*; occurring in the root system^15,94^. Apart from the four novel species described here, other *Trichoderma* species were found for the first time as endophytes in coffee: *T. atroviride*, *T. guizhouense, T. breve* and *T. theobromicola*. These species were known from other habitats, such as: tropical soils; decaying wood and bark; as mycoparasites; on mushroom compost; in leaf-cutting ant colonies; and as endophytes in *Theobroma* spp. (Malvaceae)^4,5,20,96,97^. *Trichoderma guizhouense* has a worldwide distribution, mainly in soil, and had only been reported previously as an endophyte in the endemic woody liana, *Ancistrocladus korupensis*, and in the stems of *Cola* trees in primary forest in south-west Cameroon^4^. Previously, *T. theobromicola* was known only from South America, and reported to be a common endophyte in sapwood of cacao^4,22,79^ whilst *T. breve*, a recently described species isolated from soil, was previously known only from northern China^12^. These two species are new geographical and host records for Africa, but this may simply reflect the poor sampling of *Trichoderma* in the region, particularly for endophytes. The results of the surveys also suggest that many species of *Trichoderma* are either cosmopolitan or pantropical.

Mycoparasitism—the ecological relationship where one fungus parasitizes another^98^—has now been reported for a number of species of *Trichoderma*, notably: *T. atroviride, T. hamatum, T. longibrachiatum, T. reesei* and *T. virens*, and it has recently been established that mycoparasitism is an ancestral trait of the genus^19,99^ Mycoparasitic *Trichoderma* spp. have a wide range of hosts, including true fungi, such as *Botrytis cinerea, Rhizoctonia solani, Alternaria alternata* and *Fusarium* spp., as well as Oomycetes, such as *Pythium ultimum*^49,98^. However, the species found as mycoparasites of *H. vastatrix* reported here—*T. aggressivum, T. andinense, T. parareesei, T. petersenii* and *T. pseudopyramidale*—are the first in the genus to be reported attacking the *Hemileia* rusts associated with coffee. Three of the species of *Trichoderma* obtained during the surveys are well-known mycoparasites, but were found here only growing as endophytes in coffee, namely: *T. atroviride, T. hamatum* and *T. virens*. *Trichoderma pseudopyramidale* may deserve special attention as a potential biocontrol agent of CLR, since it was the most common mycoparasitic species obtained from both Cameroon and Ethiopia (77.8% of total mycoparasites). In Ethiopia this species was commonly associated with a purported new species of *Hemileia* (cf. *H*. *coffeicola*) on wild C. *arabica* in cloud forest (ca. 2000 m). It was also frequently isolated as an endophyte from the leaves and stems of both semi-wild and wild *C*. *arabica* in Ethiopia (see Table 1). It may encompass dual roles as an endophytic bodyguard of coffee and also as a contact mycoparasite of CLR.

Mycoparasitic fungi associated with coffee rust have been studied n regions of the world where coffee is not a native species, such as in Mexico^100^. It is interesting to note that this Mexican survey identified six purported mycoparasites: *Acremonium byssoides, Calcarisporium ovalisporum*, *C. arbuscula, Fusarium pallidoroseum, Sporothrix guttuliformis* and *Verticillium* (= *Akanthomyces* *lecanii*). A more recent publication reporting the results of an investigation in Mexico and Puerto Rico, involving the use of single-molecule DNA sequencing of fungal rRNA gene barcodes to identify putative mycoparasites in pustules of *H. vastatrix*, yielded 15 fungal taxa associated with CLR, none of which belonged to *Trichoderma*^101^. Information on the ecology of the new *Trichoderma* species described here, and their role in nature, is limited because relatively few strains of each species were isolated during the survey; the exceptions being *T. botryosum* and *T. pseudopyramidale*, which constituted over 60% of the total isolations and seem to have a close association with their *Coffea* hosts, in both Cameroon and Ethiopia, in wild, semi-wild and cultivated situations.

The aim of the present study was to collect and catalogue endophytes of *Coffea* species—as well as the mycoparasites of the associated *Hemileia* rusts—in their African centres of origin, as part of a project to screen and assess these isolates as potential biological control agents of CLR. The target area is Central America where the rust has become a critical constraint to coffee production, as well as causing a socio-economic crisis, over the past decade^53,102,103^. The work presented here covers only the taxonomy with some observations on the ecology of the *Trichoderma* isolates resulting from the surveys in Africa, but these data will be pivotal for selecting candidate biocontrol agents for the potential management of *H*. *vastatrix* in the Americas.

The philosophy behind the overall project is based on the concepts of classical biological control and, in the case of CLR, on the Enemy Release Hypothesis which posits that exotic species become invasive and achieve pest status because of increased fitness in the absence of their coevolved natural enemies^104^. One solution to address the problem of invasive alien pests is to source, import and release coevolved natural enemies from the centres or regions of origin of the target species in order to reduce ‘pest’ fitness: the classical biological control strategy. This approach using fungal natural enemies, such as entomopathogens and plant pathogens, has been employed successfully to control invasive alien arthropod pests and weeds^105,106^, but never against alien plant diseases using mycoparasites. The evidence from our study indicates that there is a guild of *Trichoderma* species, potentially antagonistic to *H. vastatrix* in Africa, which could be exploited for biological control of CLR in Central America following the classical approach. There are claims that non-specific, indigenous mycoparasites; notably, *Lecanicillium lecanii*—now *Akanthomyces lecanii*^107^—can reduce the impact of CLR in the Americas^108^, but this is not evident based on the continuing rust outbreaks.

Another scenario has been suggested to further explain the invasiveness of alien plant species: the Endophyte-Enemy Release Hypothesis^109^, which posits that alien plants arrive not only without their coevolved natural enemies but also deficient in, or completely lacking, coevolved endophytes, some of which may be acting as symbionts (‘bodyguards’); protecting their hosts against adverse abiotic and biotic factors. Thus, in their absence, exotic crops thrive and alien weeds invade, with no natural enemies reducing plant fitness and fecundity and no bodyguards to ‘pay’ for protection. In crop species, the consequences can be catastrophic when coevolved natural enemies—lacking their own natural enemies, such as mycoparasites (in the case of fungal pathogens)—eventually catch up with their endophyte-deficient plant hosts. Such may be the case with *H*. *vastatrix* in Central America—and, of course, this may explain the devastating rust epiphytotics that destroyed coffee cultivation in Sri Lanka (Ceylon) in the nineteenth century, as well as in all the global regions where the rust has invaded^110^.

Thus, the ideal classical biological control agent for CLR would combine the best of both worlds in the form of an endophytic mycoparasite, and—as our results indicate—the genus *Trichoderma* contains such candidates. Potentially, these would not only be used to colonize the coffee leaf and parasitize the external rust pustules—as well as to target the invasive, intercellular mycelium of the rust—but also to bolster host defences through induced resistance^66,84,111^. There is increasing evidence that, in addition to induced resistance to diseases and pests, endophytic *Trichoderma* species confer a range of other benefits to their plant host, in particular, drought tolerance, resistance to abiotic factors such as salt stress and growth stimulation^5,21,23,27,28,30,32,36,43,71,72,81,85–87,112^.

Preliminary data, using *Trichoderma* isolates from the survey, are showing positive results in the laboratory with evidence of reduction in rust disease severity^113^ (Authors, unpublished). Greenhouse screening of four isolates of *Trichoderma* (COAD 2418, COAD 2417, COAD 2535 and COAD 2439), belonging to *T. hamatum* and *T. pseudopyramidale* sp. nov.., showed their ability to inhibit the germination of *H. vastatrix* urediniospores above 70% in vitro. Isolate COAD 2396 (*T. atroviride*) reduced the severity of the disease to less than 50% of the levels observed in the controls when applied before or simultaneously with *H. vastatrix* on coffee leaf discs. In addition, an isolate of *T. parareesei* (COAD 2482) promoted the growth and increased the biomass of tomato roots by 33% and 57%, respectively; whilst others are now showing the ability to increase drought tolerance^113^ (Authors, unpublished).

The methodology employed during the survey for the isolation of endophytes has proven to be robust. It has been emphasized previously that endophyte isolation is a method-dependent process and this will determine the quality and quantity of fungi obtained^114^. In our experience, isolating in situ—directly in the field from tree stems—or immediately after collection, eliminates or reduces contamination by many of the opportunistic endophytes and favours the slower-growing, potentially obligate endophytes. This has consistently been demonstrated not only during the present coffee survey in Africa, but also from previous surveys of wild species of *Theobroma* and *Hevea* in South American rainforests where this approach was pioneered^20,36,39^ These surveys resulted not only in the discovery of numerous new *Trichoderma* taxa—which are still being described^4,41^—but in many other taxonomic novelties, including new endophytic lineages of *Tolypocladium* and a new class of *Pezizomycotina*^115,116^. Moreover, they reveal the paucity of endophytes in cultivated exotic plants—in this instance, cacao and rubber—compared to wild populations of *Theobroma* and *Hevea* in natural ecosystems^39,41,117–119^. This has been confirmed during the present study, when a survey of coffee endophytes in four states of Brazil, failed to isolate any species of *Trichoderma*, providing compelling evidence that centres of origins or diversity of plants harbour unique guilds of endophytic *Trichoderma* species—as well as other genera—that could be exploited not only for classical biological control but also as potential reservoirs of novel metabolites.

In conclusion, our surveys in Africa for endophytes and mycoparasites associated with the genus *Coffea* and with its *Hemileia* rusts have revealed a highly diverse range of fungi, with many novel species; *Trichoderma* being just one component. Because of the relatively few countries (3) and localities (18) visited, and the restricted number of host plants sampled, this can only be viewed as a snapshot of the actual diversity of endophytes, as well as of mycoparasites, associated with *Coffea* in Africa, especially in forest ecosystems. Potentially, in Madagascar, where the diversity of the genus is richer with 59 confirmed species^120^, this still-untapped diversity could be even higher. Loss of forest habitats in Africa and Madagascar means that many of these fungi will go extinct, along with their host plants, before being described. The potential loss of such key antagonists of the CLR fungus—as well as of *Coffea* germplasm—should be cause for concern to coffee stakeholders.

## Materials and methods

### Sampling and isolation

**Figure 13:**
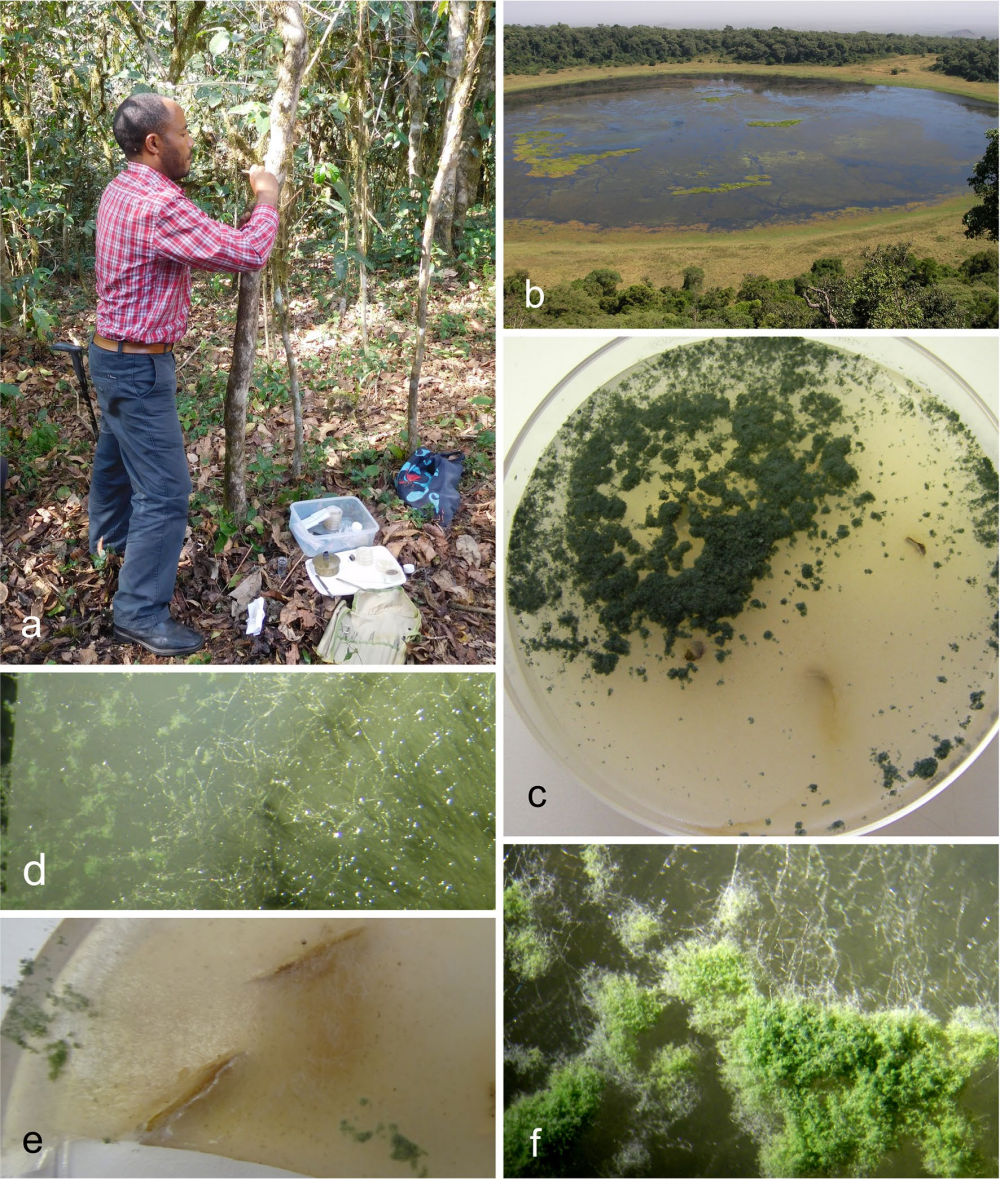
Survey protocol and isolates of *Trichoderma*. (**a**) Kifle Belachew isolating endophytes directly from main stem of *Coffea arabica* in a semi-wild coffee farm in the Kaffa Zone of Ethiopia. (**b**) Lake Paradise in Marsabit Forest Reserve, Eastern Province of Kenya where wild *Coffea* cf. *arabica* is common in the understorey. (**c**–**f**) *Trichoderma* isolates growing from Marsabit coffee stem samples, including *Trichoderma lentissimum* sp. nov. (**e**).

**Figure 14:**
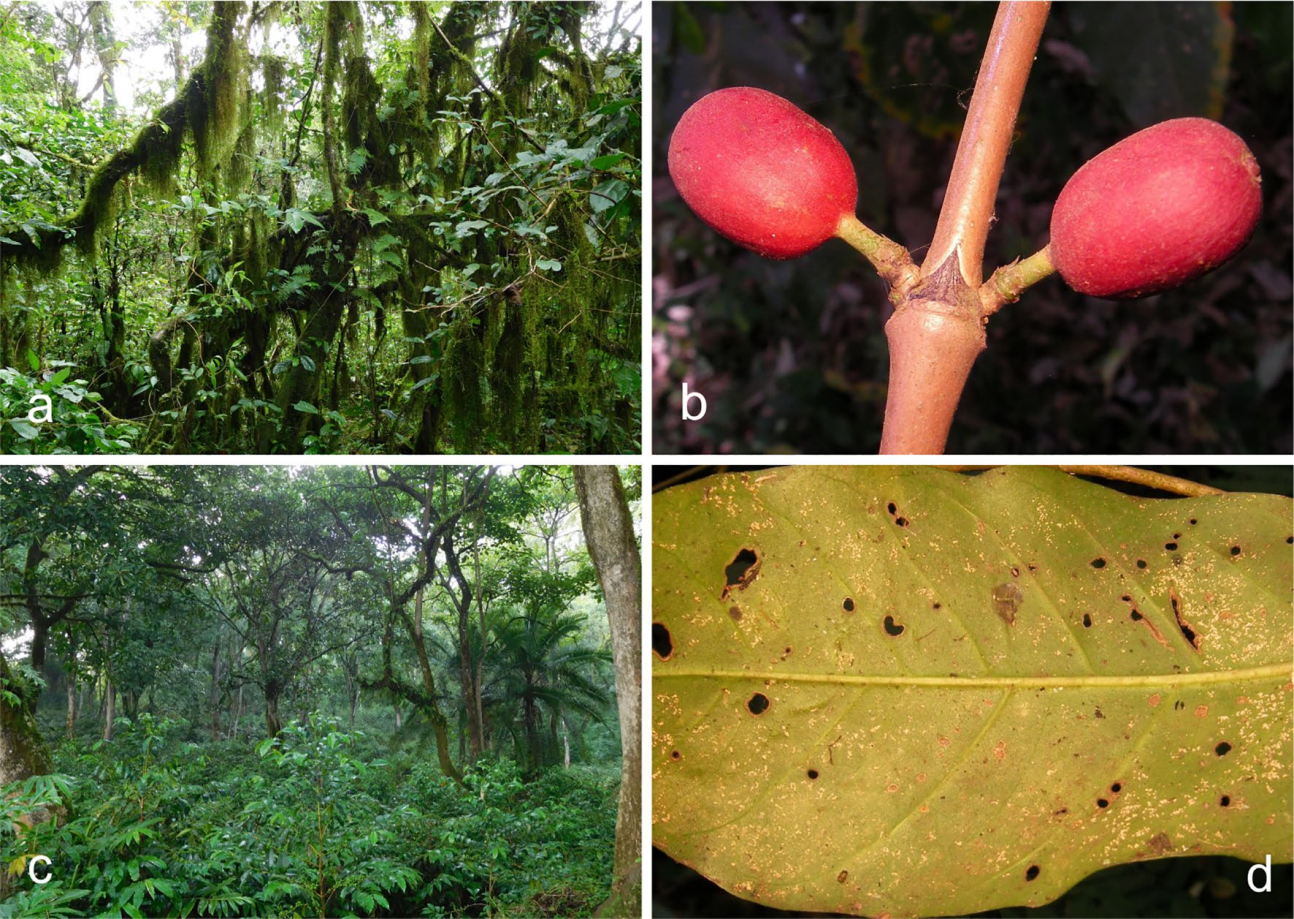
Survey in Ethiopia. (**a**) Komba Forest Reserve, Kaffa Zone, at ca. 2000 m altitude; showing wild *Coffea arabica* in the cloud forest understorey. (**b**) Wild *Coffea arabica* in Komba Forest Reserve with few, large berries at each node in contrast to the multiple, smaller berries of commercial varieties. (**c**) Typical semi-wild coffee farm of the Kaffa Zone, at ca. 1500 m altitude, established under thinned forest. (**d**) Leaf from the above wild coffee plant colonized by two rust species: the dominant white scattered pustules of *Hemileia* cf. *coffeicola*; and a typical orange dense pustule of *H*. *vastatrix*, an uncommon rust in cloud forest.

**Figure 15:**
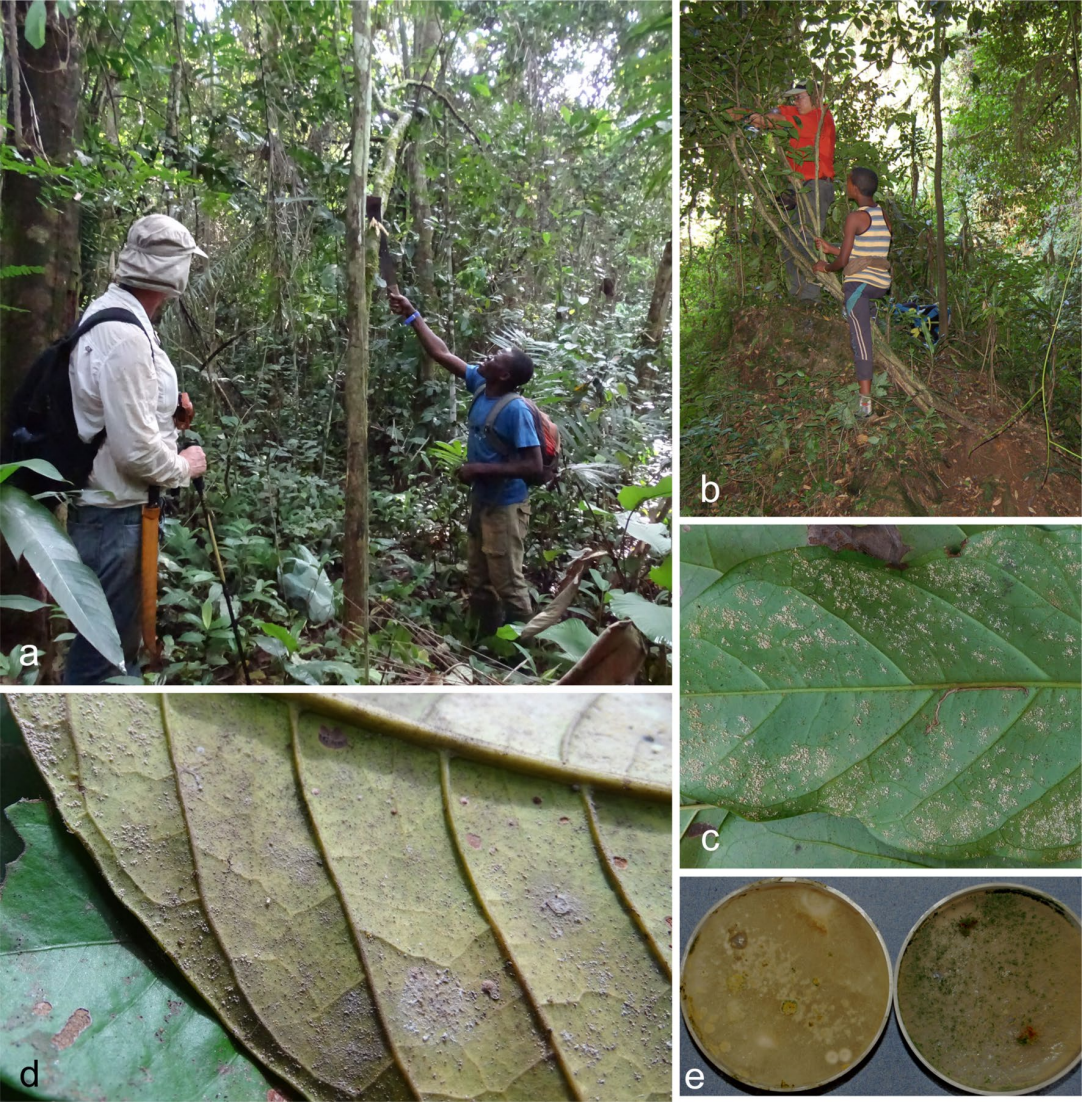
Survey sites and rust collections. (**a**) Wild *Coffea canephora* tree (> 10 m high) in understorey of Dja Forest Reserve, Eastern Province, Cameroon, ca. 700 m altitude; stem samples from this tree yielded four *Trichoderma* spp. (**b**) Collecting samples from wild *Coffea arabica* at Natural Bridge, Makira-Grugguto, Kaffa Zone, Ethiopia, ca. 1600 m altitude; *Trichoderma pseudopyramidale* sp. nov. was isolated from the leaves and stem of this tree. (**c**) *Hemileia coffeicola* on leaf of *Coffea canephora*, Somalomo Town, Eastern Province, Cameroon; showing the white to pale yellow, loose and scattered pustules. (**d**) *Hemileia coffeicola* completely parasitized by a guild of mycoparasites, including *Trichoderma pseudopyramidale* sp. nov., in a coffee plantation near Somalomo Town; imparting a grey appearance to the rust infection. (**e**) Two plates showing predomination of *Trichoderma* colonies emerging from field samples.

The fungal isolates were all obtained during survey collections in Africa, namely: Kenya (May–June, 2015); Cameroon (November, 2015); Ethiopia (November, 2015; May–June, 2017; January, 2018). In addition, surveys were made in coffee farms in Brazil to compare and contrast the guilds of *Trichoderma* present in the native and exotic ranges of coffee. Surveys were undertaken in cooperation with African scientists from local research organizations: notably, IRAD (Institut de Recherche Agricole pour le Developpement), in Cameroon; Jimma University and Ethiopian Institute of Agricultural Research, in Ethiopia. Ad hoc surveys were also undertaken by the local scientists. The surveys were targeted at areas where wild species of *Coffea* occur and, specifically, where the main species of commerce—*Coffea arabica* (Kenya and Ethiopia) and *Coffea canephora* (Cameroon—Congo Basin)—are present in the wild, or are cultivated in semi-wild conditions (Figs. 13a,b, 14a–c, 15a,b). At each selected site, *Coffea* plants were examined for rust pustules—with particular attention to collecting rust colonies exhibiting mycoparasitism, or appearing to be abnormal (unusual colour, poor sporulation) (Figs. 14d, 15c,d). Specimens were dried in a plant press for later processing in the laboratory (preliminary identification and isolation). Also, at each site, samples of at least three separate adult plants were collected, consisting of healthy leaves, berries and 3-cm diam or thicker stem sections of each individual, and bagged for examination and processing later the same day. Isolations were made from healthy leaves, stems and berries of *C*. *arabica, C. brevipes*, *C. canephora* and *C. eugenioides* (Figs. 13c–f, 15e). The isolation protocol followed the procedure described by Evans et al.^20^ with modifications, and were performed as described below.

1. Stems in situ were thoroughly rubbed with cotton wool soaked in 70% alcohol and, after the alcohol had evaporated, the bark was removed using a flamed knife or machete blade (Fig. 13a). The exposed panel was then cleaned with a scalpel (Swann Morton 10) and the surface further pared with a smaller blade (Swann Morton 11). Nine, triangular slivers of sapwood (*ca*. 8 × 5 mm) were excised with a scalpel (Swann Morton 10A) from the panel and transferred individually with fine forceps to three plastic Petri plates (3- or 5-cm diam; 3 samples/plate), containing selective media: potato dextrose agar (PDA), one-fifth strength (20% PDA), supplemented with 10 mg/l penicillin–streptomycin solution. These were sealed immediately with electrical tape and stored in plastic boxes. During these procedures, all instruments were surface sterilized in 90% ethanol and flamed using a portable, alcohol burner. On arrival at the laboratory, the plates were transferred to a 25 °C incubator and examined regularly over an 8-week period. Hyphal tips or spores were excised or picked from colonies as they appeared on or around the wood samples and transferred to 5-cm diam, plastic Petri plates containing 20% PDA or potato carrot agar (PCA) and incubated under black light at 25 °C to promote sporulation. This procedure was firstly described for the isolation of endophytic fungi by Evans et al.^20^ but was applied here for the first time for endophytic fungi from coffee.
2. Young mature healthy leaves (third from the branch tip) were thoroughly rubbed with cotton wool soaked in 70% alcohol and, after the alcohol had evaporated, three small (*ca*. 5 × 5 mm) square fragments were excised from the leaf centre (including the midrib) and were surface sterilized for 3 min by immersion in 10% bleach, followed by immersion in sterile water in stoppered plastic tubes and, following a thorough agitation, were plated as described for stems. Subsequent processing was as described for stems.
3. Whenever available, *Coffea* berries were also sampled and treated similarly as described above for leaf samples but, after surface cleaning with alcohol, each fruit was skinned and inner parts were divided into three slices which were then surface sterilized before plating. Further steps followed the same procedure as described above.

For the isolation of mycoparasites, conidia from parasitized rust pustules were selected and picked-off with a sterile needle, using a dissecting microscope, and transferred to PDA plates. The dried samples were processed within 2 weeks of collection after transport to the laboratory in the UK or Brazil.

**Figure 16:**
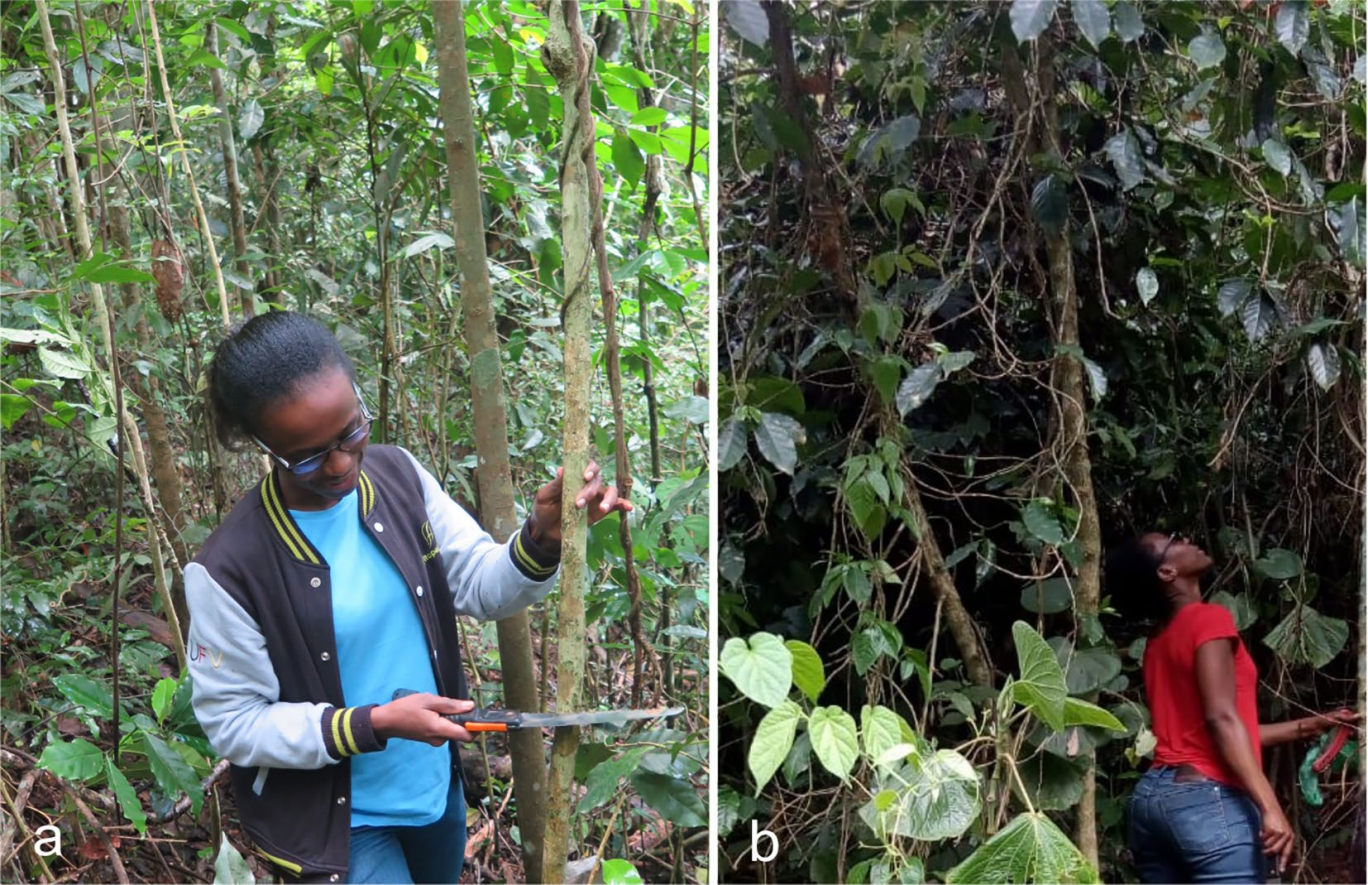
Survey sites in semi-wild situations in Brazil. (**a**) Miraine Ndacnou collecting a sample of mature *Coffea arabica* from the forest reserve Mata do Paraíso (Universidade Federal de Viçosa, state of Minas Gerais, Brazil). Notice the well-established secondary forest (Mata Atlântica) under which the coffee plants exist. (**b**) Miraine Ndacnou selecting a mature *Coffea arabica* growing in a forest fragment (Mata Atlântica) of the Fazenda Camocim (Pedra Azul, Domingos Martins, state of Espírito Santo, Brazil) for sampling.

The same endophyte isolation protocol described above was utilized for samples collected at eight localities in four Brazilian states (Espírito Santo, Minas Gerais, São Paulo and Rio de Janeiro). Survey sites closest to those where coffee was sampled in Africa were selected; concentrating on those where coffee plants were growing in semi-wild or forest situations, such as abandoned coffee farms and invasive populations in Atlantic rainforest (Fig. 16).

### DNA extraction, PCR amplification and sequencing

Strains were grown in 3-cm diam plates containing 5 mL of potato dextrose broth (PD) at 25 °C in the dark for 4–5 days. DNA was extracted from the mycelium grown on the surface of the broth. DNA was extracted with the Wizard Genomic DNA Purification kit (Promega, Madison, EUA) by following the manufacturer’s instructions. The fragments *rpb2* (primers fRPB27cR—RPB25F2)^121^ and *tef1* (primers EF2—EF1728M) were amplified for all isolates and additionally *cal* (primers CAL228–CAL737)^122^ was amplified for a subset of 12 isolates.

The polymerase chain reaction (PCR) amplifications were performed in a total reaction volume of 12.5 μl, including 0.25 μl of each primer, 1.25 µl of BSA, 6.25 of Taq polymerase [including dNTPs], 0.25 µl of genomic DNA [30 ng/µl]; 0.25 µl DMSO and 4 µl of sterile ultrapure water. PCR conditions for *rpb2* were 95 °C/5 min., followed by 38 cycles at 95 °C/1 min., 58 °C/2 min., 72 °C/2 min. and 72 °C/10 min. For *tef1*, conditions were 94 °C/2 min., followed by 9 cycles at 94 °C/35 s, 66 °C/ 55 s, and 35 cycles at 94 °C/35 s, 56 °C/55 s and 72 °C/1 min 30 s. Conditions for *cal* were 95 °C/8 min., followed by 35 cycles at 95 °C/15 s, 55 °C/20 s, 72 °C/1 min and extension at 72 °C/5 min. PCR products were visualized by Gelred (Thermo Fisher Scientific) staining following electrophoresis of 4 μl of each product in 1% agarose gel. The PCR products were sequenced by Macrogen Inc., South Korea (http://www.macrogen.com).

### Phylogenetic analysis

Consensus sequences were assembled from forward and reverse sequencing chromatograms using SeqAssem^123^ *tef1*, *rpb2* and *cal* contigs of all strains were compared to homologous sequences deposited in NCBI GenBank. Sequences generated in the present study were deposited in the NCBI GenBank database (Table 1) and sequences obtained in other studies were used in our phylogenetic analyses and were retrieved from the NCBI GenBank database (Supplementary Table S1) T. Sequence alignments were performed using MUSCLE implemented in MEGA 10^124^. In total, the dataset comprised 324 partial *tef1* (sequences 664 pb); 169 partial *rpb2* sequences (951 pb) and 25 partial *cal* sequences (443 pb). Two concatenated trees with *tef1* and *rpb2* sequences were created, one with taxa of the clades *Harzianum* (more numerous), *Stricpile* and *Virens*, and the other with the rest of the taxa (Figs. 1 and 2); a third concatenated analysis with partial sequences of three genes*, tef, rpb2* and *cal*, was constructed with a subgroup of sequences to clarify thephylogenetic relationships of some species within the clade *Harzianum* (Fig. 3), such trees containing 168 taxa with 2515 characters, 86 taxa with 2422 characters and 25 taxa with 1927 characters, respectively. The concatenated alignments were generated in Sequence matrix v1.8^125^. Single-gene trees were also generated. Maximum parsimony (MP), Maximum likelihood (ML) and Bayesian Inference (BI) were performed for the concatenated and single-gene trees. Prior to phylogenetic analyses, the most appropriate nucleotide substitution model for each locus was selected using MRMODELTEST v.2^126^. Nucleotide substitution models in the two-gene concatenated trees were HKY + I + G and SYM + I + G (Figs. 1 and 2), for *tef1* and *rpb2*, respectively. For the three-gene concatenated tree, the models were HKY + I, K80 + I and K80 + G (Fig. 3) for *tef*, *rpb2* and *cal*, respectively. For all trees the BI and ML analysis were estimated in the CIPRES Science Gateway Platform using Mr. Bayes 3.2.6 and RaxML-HPC v.8, respectively^127,128^ and MP in MEGA 10. Phylogenetic trees were visualized using FigTree (http://tree.bio.ed.ac.uk/software/figtree/). Phylogenetic species were recognized based on two main previously accepted criteria^129^ Genealogical Concordance (the clade was present in the majority of the single-locus genealogies, as revealed by a majority-rule consensus tree) and Genealogical Non-discordance (the clade was well supported in the least one single-locus genealogy, as judged both by MP and BI and was not contradicted in any other single-locus genealogy at the same level of support).

### Morphological characteristics

The results of the phylogenetic analysis of the assemblage of *Trichoderma* isolates guided the selection of isolates to be included in the morphological analysis and characterization of novel taxa. One or two isolates of each new taxon were examined. Procedures for morphological observation of *Trichoderma* followed the protocol established by Samuels and Hebbar^5^. Macroscopic characteristics of colonies—mycelium colour, radial growth, presence/absence of concentric rings, sporulation “pustules”^62^, pigmentation and presence/absence of odour—were evaluated on PDA, CMD (Corn-meal Agar) and SNA (Synthetic Nutrient Deficient Agar) after 7 days at 25 °C under a 12-h daily light regime (light provided by two white and one near-UV lamps placed 35 cm above the plates). Rates of growth were evaluated at 72 and 96 h on the three culture media at 25, 30 and 35 °C in the dark. Observations of fungal structures were made using an Olympus BX 51 microscope and were based on slide cultures prepared with colonies of each isolate growing from PDA and CMD blocks^130^. After 4–5 days of growth at 25 °C under the same light regime described above, the slides were mounted in 3% KOH for observation and illustration. Descriptions included biometric data of phialides, conidia and chlamydospores. Measurements were taken from images generated with a digital camera Olympus Q-Color 3.

### Statement

All experimental protocols adopted during this research were approved by the Comissão de Pesquisa do Departamento de Fitopatologia—Universidade Federal de Viçosa.

### Informed consent

Informed consent was obtained to publish the names/information/images of all study participants appearing in the publication.

### Ethical approval

All experimental protocols were approved by a named institutional and/or licensing committee/s. All methods were carried out in accordance with relevant guidelines and regulations.

## Supplementary Information

Supplementary Table S1.
