# Author Correction: New species and records of *Trichoderma* isolated as mycoparasites and endophytes from cultivated and wild coffee in Africa

**DOI:** 10.1038/s41598-021-97704-7

**Published:** 2021-09-22

**Authors:** María del Carmen H. Rodríguez, Harry C. Evans, Lucas M. de Abreu, Davi M. de Macedo, Miraine K. Ndacnou, Kifle B. Bekele, Robert W. Barreto

**Affiliations:** 1 Departamento de Fitopatologia, Universidade Federal de Viçosa, Viçosa, MG 36570‑900 Brazil; 2 CAB International, Bakeham Lane, Egham, Surrey TW20 9TY UK; 3 IRAD-Institut de Recheche Agricole pour le Developpement, BP 2067, Yaoundé, Cameroon; 4 Department of Horticulture and Plant Science, College of Agriculture and Veterinary Medicine, Jimma University, P.O. Box 397, Jimma, Ethiopia; 5 Ethiopian Institute of Agriculture Research, P.O. Box 192, Jimma, Ethiopia

Correction to: *Scientific Reports* https://doi.org/10.1038/s41598-021-84111-1, published online 11 March 2021

The original version of this Article contains errors.

The cultures designated as holotypes of *Trichoderma botryosum*, *Trichoderma caeruloviride*, *Trichoderma lentissimum* and *Trichoderma pseudopyramidale* were not indicated to be in a metabolically inactive state. As a consequence, the proposed new species are invalid under the Shenzhen Code (Turland et al. 2018)^1^.

Those species are validated herein:

***Trichoderma botryosum*** M.C.H. Rodríguez, H.C. Evans & R.W. Barreto **sp. nov.** –MycoBank: MB840985

For a detailed description see: Rodríguez *et. al.*, *Sci Rep* **11** (no. 5671): page 12 (2021)

*Holotype.* ETHIOPIA: Southern Nations, Nationalities and Peoples Region, Kaffa Zone, Bonga District, Gela Wild Coffee Biosphere Reserve, cloud forest, alt 1900 m; isolated as an endophyte from berries of *Coffea arabica*; 25 November 2015, H.C. Evans, K. Belachew & R.W. Barreto. VIC 47493 (dried metabolically inactive culture). Ex-type culture: COAD 2422.

***Trichoderma caeruloviride*** M.C.H. Rodríguez, H.C. Evans & R.W. Barreto, **sp. nov.** –Mycobank: MB840986

For a detailed description see: Rodríguez *et. al.*, *Sci Rep* **11** (no. 5671): page 14 (2021)

*Holotype*: ETHIOPIA: Southern Nations, Nationalities and Peoples Region, Kaffa Zone, Bonga District, Gedam Village, coffee farm, alt 1550 m; isolated as an endophyte from berries of *Coffea arabica*. 25 November 2015, H.C. Evans, K. Belachew & R.W. Barreto. VIC 47494 (dried metabolically inactive culture). Ex-type culture: COAD 2415.

***Trichoderma lentissimum*** M.C.H. Rodríguez, H.C. Evans & R.W. Barreto **sp. nov.** – Mycobank: MB840987

For a detailed description see: Rodríguez *et. al.*, *Sci Rep* **11** (no. 5671): page 16 (2021)

*Holotype*: KENYA: Eastern Province, Marsabit National Park, Lake Paradise, primary forest, alt 1340 m; isolated as an endophyte from stem of *Coffea* cf. *arabica*, 28 January 2015, H.C. Evans & R.W. Barreto. VIC 47495 (dried metabolically inactive culture). Ex-type culture: COAD 2399.

***Trichoderma pseudopyramidale*** M.C.H. Rodríguez, H.C. Evans & R.W. Barreto **sp. nov.** – Mycobank: MB840988

For a detailed description see: Rodríguez *et. al.*, *Sci Rep* **11** (no. 5671): page 18 (2021)

*Holotype*: ETHIOPIA: Southern Nations, Nationalities and Peoples Region, Kaffa Zone, Bonga District, Maakira-Grugutto, semi-wild coffee farm, alt 1600 m; isolated as an endophyte from leaves and stems of *Coffea arabica*, 25 November 2015, H.C. Evans & R.W. Barreto. VIC 477496 (dried metabolically inactive culture). Ex-type culture: COAD 2426.

The authors apologize for any inconvenience caused.
