# Supplementary material for: New species and records of Trichoderma isolated as mycoparasites and endophytes from cultivated and wild coffee in Africa
Source: Sci Rep. 2021 Mar 11;11:5671. doi: 10.1038/s41598-021-84111-1 (PMC7952591; doi:10.1038/s41598-021-84111-1)
Supplement: Supplementary file 1 — Supplementary Table S1. [file 41598_2021_84111_MOESM1_ESM.docx]

# ****New species and records of****Trichoderma****isolated as mycoparasites and endophytes from cultivated and wild coffee in Africa****

**María del Carmen H. Rodríguez, Harry C. Evans, Lucas M. de Abreu, Davi M. de Macedo, Miraine K. Ndacnou, Kifle B. Bekele, Robert W. Barreto**

**Table S1.** Trichoderma from NCBI GenBank accessions used in the phylogenetic analyses.

|  | **GenBank accessions** | | |  |  |
| --- | --- | --- | --- | --- | --- |
| **Taxon** | **Strain** | **Country** | ***tef1*** | ***rpb2*** | ***cal*** |
| *T. adaptatum* | HMAS 248800 | China | KX428024 | KX428042 | — |
| *T. adaptatum* | HMAS 248801 | China | KX428027 | KX428045 | — |
| *T. afarasin* | DIS 377A | — | FJ463322 | FJ442799 | — |
| *T. afarasin* | DIS 314F | — | FJ463400 | FJ442778 | — |
| ***T. afroharzianum*** | **CBS 124620 = G.J.S. 04-186** | **Peru** | **FJ463301** | **FJ442691** | **FJ442370** |
| *T. afroharzianum* | G.J.S. 00-24 | México | AF443940 | FJ442726 | AF442880 |
| *T. afroharzianum* | LESF229 | Brazil | KT279013 | KT278945 | — |
| *T. aggregatum* | HMAS 248863 | China | KY688062 | KY688001 | — |
| *T. aggregatum* | HMAS 248864 | China | KY688063 | KY688002 | — |
| *T. aggressivum* | DAOM 222156 | Canada | AF348098 | FJ442752 | — |
| ***T. aggressivum*** | **CBS 100526 (T), CBS 100525; CBS 100525** | **Ireland; England** | **AF348096** | **AF545541** | **—** |
| *T. alpinum* | HMAS 248821 | China | KY688012 | KY687958 | **—** |
| *T. alpinum* | HMAS 248870 | China | KY688017 | KY687963 | **—** |
| *T. alni* | Hypo 254 = CBS 120633 (T) | England | EU498312 | EU498349 | — |
| *T. amazonicum* | IB 95 | Peru | HM142377 | HM142368 | — |
| *T. andinense* | DAOM 220821 | Venezuela | EU280042 | KJ842208 | — |
| *T. andinense* | GJS 09-62 | Peru | JN133570 | JN175533 | — |
| *T. andinense* | LESF541 | Brazil | KT279037 | KT278979 | — |
| ***T. andinense*** | **G.J.S. 90-140 = CBS 354.97 = ATCC 208857 (T)** | **Venezuela** | **AY956321** | **JN175531** | **—** |
| *T. asperelloides* | G.J.S. 04-116 | Vietnam | GU248412 | GU248411 | — |
| ***T. asperellum*** | **CBS 433.97 = TR3 (T)** | **USA** | **AF456907** | **EU248617** | **—** |
| *T. atrobrunneum* | S3 | Italy | KJ665376 | KJ665241 | — |
| *T. atrobrunneum* | CBS 130440 = G.J.S. 04-67 | Italy | FJ463360 | FJ442724 | FJ442329 |
| *T. atrobrunneum* | GJS 05-101 | — | FJ463392 | FJ442745 | FJ442331 |
| *T. atrobrunneum* | CBS 548.92 = G.J.S. 92-110 | France | — | — | AF442883 |
| *T. atroviride* | CBS 119499 | Austria | FJ860611 | FJ860518 | — |
| *T. atroviride* | DAOM 222144 | Canada | AF456889 | FJ442754 | — |
| ***T. atroviride*** | **CBS 142.95** | **Slovenia** | **AY376051** | **EU341801** | **—** |
| *T. atroviride* | Th002 | Colombia | AB558906 | AB558915 | — |
| *T. azevedoi* | CEN1403 | Brazil | MK696638 | MK696800 | **—** |
| *T. azevedoi* | CEN1422 | Brazil | MK696660 | MK696821 | **—** |
| *T. bannaense* | HMAS:248840 | China | KY688037 | KY687979 | **—** |
| *T. bannaense* | HMAS 248865 | China | KY688038 | KY688003 | **—** |
| *T. beijingense* | HMAS 248804 | China | KX428025 | KX428043 | — |
| *T. beijingense* | HMAS 248805 | China | KX428026 | KX428044 | — |
| *T. bifurcatum* | HMAS 248795 | China | KX428018 | KX428036 | — |
| *T. breve* | HMAS 248844 | China | KY688045 | KY687983 | **—** |
| *T. breve* | HMAS 248845 | China | KY688046 | KY687984 | **—** |
| ***T. brunneoviride*** | **Hypo 170 = CBS 121130; Hypo 442 = CBS 120928** | **—** | **EU498316** | **EU498358** | **—** |
| *T. camerunense* | GJS 99-230 | — | AF348107 | — | — |
| *T. camerunense* | GJS 99-231 | — | AF348108 | — | — |
| *T. caribbaeum* | CBS 119093 = G.J.S. 97-3 (T) | Guadeloupe | KJ665443 | KJ665246 | — |
| *T. catoptron* | G.J.S. 02-76 = CBS 114232 (T) | Sri Lanka | AY737726 + AY391963 | AY391900 | — |
| *T. compactum* | CBS 121218 | — | KF134798 | KF134789 | — |
| *T. concentricum* | HMAS 248833 | China | KY688027 | KY687971 | **—** |
| *T. concentricum* | HMAS 248858 | China | KY688028 | KY687997 | **—** |
| *T. dorotheae* | G.J.S. 99-202 (T) | New Zealand | DQ307536 | EU248602 | — |
| *T. endophyticum* | CBS 130733 =DIS 220j | Ecuador | FJ463330 | FJ442690 | — |
| *T. endophyticum* | DIS 220k | Ecuador | FJ463328 | FJ442765 | — |
| *T. endophyticum* | DIS 221e | Ecuador | FJ463316 | FJ442775 | — |
| *T. epimyces* | CPK 1980 | — | EU498319 | EU498359 | — |
| *T. evansii* | DIS341hi = CBS 123079 (T) | Ecuador | EU883566 | EU883558 | — |
| *T. gamsii* | G.J.S. 04-09 | USA | DQ307541 | JN133561 | — |
| ***T. ghanense*** | **G.J.S. 95-137 = IAM 13109 (T)** | **Ghana** | **AY937423** | **JN175559** | **—** |
| *T. ghanense* | DAOM 165776 | USA | JN175610 | JN175560 | — |
| *T. gracile* | CBS 130714 = G.J.S. 10-263 (T) | Malaysia | JN175598 | JN175547 | — |
| *T. guizhouense* | S278 | Croatia | KF134799 | KF134791 | — |
| *T. guizhouense* | LESF554 | Brazil | KT279017 | KT278952 | — |
| *T. guizhouense* | S628 | Greece | KJ665511 | KJ665273 | — |
| ***T. guizhouense*** | **HGUP 0038 = CBS 131803** | **China** | **JN215484** | **JQ901400** | **—** |
| ***T. hamatum*** | **DAOM 167057 (T)** | **Canada** | **EU279965** | **AF545548** | **—** |
| *T. hamatum* | Hypo 647 | France | KJ665513 | KJ665274 | — |
| *T. hamatum* | Hypo 648 = CBS 132565 | France | KJ665514 | KJ665275 | — |
| *T. harzianum* | GJS 04-71 | — | FJ463396 | FJ442779 | — |
| *T. harzianum* | GJS 05-107 | — | FJ463329 | FJ442708 | — |
| *T. harzianum* | CBS 227.95 | UK | AF348100 | — | — |
| ***T. harzianum*** | **CBS 226.95** | **UK** | **AF348101** | **AF545549** | **—** |
| *T. hausknechtii* | Hypo 649 = CBS 133493 (T) | France | KJ665515 | KJ665276 | — |
| *T. hengshanicum* | HMAS 248852 | China | KY688054 | KY687991 | — |
| *T. hengshanicum* | HMAS 248853 | China | KY688055 | KY687992 | — |
| *T. hirsutum* | HMAS 248834 | China | KY688029 | KY687972 | — |
| *T. hirsutum* | HMAS 248859 | China | KY688030 | KY687998 | — |
| *T. ingratum* | HMAS 248822 | China | KY688018 | KY687973 | — |
| *T. ingratum* | HMAS 248873 | China | KY688022 | KY688010 |  |
| *T. istrianum* | S310 = CBS 130539 (T) | Croatia | KJ665523 | KJ665281 | — |
| *T. italicum* | S131 = CBS 132567 (T) | Italy | KJ665525 | KJ665282 | — |
| *T. junci* | CBS 120926 = Hypo 399 (T) | Denmark | FJ860641 | FJ860540 | — |
| *T. koningiopsis* | S359 | France | KJ665546 | KJ665285 | — |
| *T. koningiopsis* | LESF212 | USA | KT278985 | KT278914 | — |
| ***T. koningiopsis*** | **G.J.S. 93-20 (T)** | **Cuba** | **DQ284966** | **EU241506** | **—** |
| *T. lentiforme* | DIS 173f | Brazil | FJ463347 | FJ442787 | — |
| *T. lentiforme* | DIS 167e | Brazil | FJ463333 | FJ442764 | FJ442366 |
| *T. lentiforme* | DIS 167C | Brazil | FJ463309 | FJ442689 | FJ442365 |
| *T. lentiforme* | DIS 218e | Ecuador | FJ463310 | FJ442793 | — |
| *T. lieckfeldtiae* | G.J.S. 00-14 = CBS 123049 (T) | Colombia | EU856326 | EU883562 | — |
| *T. liberatum* | HMAS 248831 | China | KY688025 | KY687969 | **—** |
| *T. liberatum* | HMAS 248832 | China | KY688026 | KY687970 | **—** |
| *T. linzhiense* | HMAS 248846 | China | KY688047 | KY687985 | **—** |
| *T. linzhiense* | HMAS 248874 | China | KY688048 | KY688011 | **—** |
| *T. lixii* | C.P.K. 1724 = G.J.S. 05-32 | Cameroon | EF191328 | — | — |
| *T. lixii* | C.P.K. 1720 = G.J.S. 05-82 | Cameroon | EF191326 | — | — |
| ***T. lixii*** | **G.J.S. 97-96 = CBS 110080 = C.P.K. 2784 (T epi)** | **Thailand** | **FJ716622** | **KJ665290** | **—** |
| *T. mangshanicum* | HMAS 248810 | China | KX428032 | KX428050 | **—** |
| *T. mangshanicum* | HMAS 248811 | China | KX428033 | KX428051 | **—** |
| ***T. neocrassum*** | **DAOM 164916 = CBS 336.93 = C.P.K. 63 (T ana)** | **Canada** | **EU280048** | **AF545542** | **—** |
| *T. neokoningii* | G.J.S. 04-216 = CBS 120070 (T) | Peru | KJ665620 | KJ665318 | — |
| ***T. parareesei*** | **CBS 125925, TUB F-1066** | **Mexico** | **GQ354353** | **HM182963** | **—** |
| *T. parareesei* | G.J.S. 07-26 | Ghana | GQ354373 | HM182966 | — |
| *T. parareesei* | G.J.S. 04-41 | Brazil | GQ354372 | HM182964 | — |
| *T. parareesei* | TUB F-430 | Sri Lanka | GQ354351 | HM182968 | — |
| *T. paratroviride* | S385 = CBS 136489 (T) | Spain | KJ665627 | KJ665321 | — |
| *T. paucisporum* | G.J.S. 01-13 = CBS 118645 (T) | Ecuador | DQ109540 | FJ150787 | — |
| *T. peberdyi* | CEN1387 | Brazil | MK696619 | MK696781 |  |
| *T. peberdyi* | CEN1388 | Brazil | MK696620 | MK696782 |  |
| *T. petersenii* | S200 | Portugal | KJ665636 | KJ665327 | — |
| ***T. petersenii*** | **CBS 119507 = Hypo 45** | **Austria** | **FJ860670** | **FJ860568** | **—** |
| *T. petersenii* | G.J.S. 04-164 | USA | DQ289004 | FJ442783 | — |
| ***T. pleuroticola*** | **CBS 124383 (T)** | **Korea** | **HM142381** | **HM142371** | **—** |
| *T. pleuroticola* | T1295 | — | EU279973 | — | — |
| *T. pleurotum* | CBS 124387 (T) | Korea | HM142382 | HM142372 | — |
| *T. polypore* | HMAS 248855 | China | KY688058 | KY687994 | **—** |
| *T. polypore* | HMAS:248861 | China | KY688059 | KY688000 | **—** |
| *T. priscilae* | S129 | Italy | KJ665689 | KJ665332 | — |
| *T. pseudodensum* | HMAS:248828 | China | KY688023 | KY687967 | **—** |
| *T. pseudodensum* | HMAS:248829 | China | KY688024 | KY687968 | **—** |
| *T. pubescens* | DAOM 166162 (T) | USA | AY750887 | EU248613 | — |
| *T. pyramidale* | S119 | Italy | KJ665696 | — | — |
| ***T. pyramidale*** | **S573** | **Italy** | **KJ665698** | **—** | **—** |
| *T. pyramidale* | S73 = CBS 135574 (T) | Italy | KJ665699 | KJ665334 | — |
| *T. pyramidale* | S533 | Spain | KJ665697 | — | — |
| *T. reesei* | G.J.S. 00-89 | Brazil | JN175599 | JN175548 | — |
| *T. reesei* | G.J.S. 00-09 | Mexico | JN175600 | JN175549 | — |
| *T. reesei* | G.J.S. 06-138 | Cameroon | GQ354370 | HM182972 | — |
| ***T. reesei*** | **QM 6a** | **Solomon Islands** | **Z23012** | **HM182969** | **—** |
| *T. rifaii* | DIS 337f | — | FJ463321 | FJ442720 | — |
| *T. rifaii* | DIS 355b | — | FJ463324 | — | — |
| *T. scalesiae* | G.J.S. 03-74 (T) | Ecuador | DQ841726 | EU252007 | — |
| *T. shaoguanicum* | HMAS 248808 | China | KX428030 | KX428048 | — |
| *T. shaoguanicum* | HMAS 248809 | China | KX428031 | KX428049 | — |
| *T. simmonsii* | S7 | Italy | KJ665719 | KJ665337 | — |
| *T. simmonsii* | CBS 123765 = G.J.S. 90-127 | USA | AF443936 | FJ442798 | — |
| *T. simmonsii* | CBS 123799 = IMI 393966 = G.J.S. 90-22 | USA | AF443933 | AY391925 | — |
| *T. simplex* | HMAS 248842 | China | KY688041 | KY687981 | **—** |
| *T. simplex* | HMAS 248860 | China | KY688042 | KY687999 | **—** |
| *T. solum* | HMAS 248848 | China | KY688050 | KY687987 | **—** |
| *T. solum* | HMAS 248849 | China | KY688051 | KY687988 | **—** |
| *T. spirale* | S212 | Spain | KJ665740 | KJ665348 | — |
| *T. spirale* | LESF117 | Brazil | KT279024 | KT278958 | — |
| *T. spirale* | LESF107 | Brazil | KT279022 | KT278956 | — |
| ***T. spirale*** | **DAOM 183974; DIS 311d** | **—** | **EU280049** | **FJ442694** | **—** |
| *T. stilbohypoxyli* | Hypo 256 = C.P.K. 1977 | England | FJ860702 | FJ860592 | — |
| *T. stramineum* | G.J.S. 02-84 |  | AY737746 | AY391945 | — |
| *T. tardum* | HMAS 248798 | China | KX428020 | KX428038 | **—** |
| *T. tardum* | HMAS 248799 | China | KX428021 | KX428039 | **—** |
| ***T. theobromicola*** | **DIS 85f (T)** | **Peru** | **EU856321** | **FJ007374** | **—** |
| *T. theobromicola* | DIS 376f (T) | Cameroon | — | FJ150786 | — |
| *T. tomentosum* | CBS 120637 = C.P.K. 2498 | Austria | FJ860629 | FJ860532 | — |
| *T. tomentosum* | S33 | — | KF134801 | KF134793 | — |
| ***T. velutinum*** | **DAOM 230013; C.P.K. 298** | **—** | **AY937415** | **KF134794** | **—** |
| *T. virens* | DIS 328a | Ecuador | FJ463363 | FJ442738 | — |
| *T. virens* | DIS 162 | Costa Rica | FJ463367 | FJ442696 | — |
| *T. virens* | CBS 123790 = G.J.S. 01-287 | Ivory Coast | AY750894 | EU341804 | — |
| *T. vulgatum* | HMAS 248796 | China | KX428019 | KX428037 | — |
| *T. vulgatum* | HMAS 248797 | China | KX428035 | KX428053 | — |
| *T. zayuense* | HMAS 248835 | China | KY688031 | KY687974 | **—** |
| *T. zayuense* | HMAS 248836 | China | KY688032 | KY687975 | **—** |
| *Protocrea pallida* | CBS 121552 = Hypo 376; CBS 299.78 (T) | Denmark | EU703900 | EU703944 | — |

*Trichoderma* strains isolated during this study; ex-type strains are indicated in bold.
